# Immunity to *Streptococcus pyogenes* and Common Respiratory Viruses at Age 0 to 4 Years After COVID-19 Restrictions

**DOI:** 10.1001/jamanetworkopen.2025.37808

**Published:** 2025-10-15

**Authors:** Kitt Dokal, Samuel Channon-Wells, Catherine Davis, Diego Estrada-Rivadeneyra, Kristin K. Huse, Amelia Lias, Shea Hamilton, Rebecca L. Guy, Theresa Lamagni, Sam Nichols, Andrew Taylor, Philipp K. A. Agyeman, Amutha Anpananthar, Romain Basmaci, Enitan D. Carrol, Michael J. Carter, Tisham De, Marien I. de Jonge, Marieke Emonts, Leire Estramiana Elorrieta, Katy Fidler, Mojca Kolnik, Taco W. Kuijpers, Federico Martinon-Torres, Henriette Moll, Marine Mommert-Tripon, Samira Neshat, Maggie Nyirenda-Nyang’wa, Sean O’Riordan, Daniel R. Owens, Nazima Pathan, Stephane Paulus, Mark J. Peters, Marko Pokorn, Andrew J. Pollard, Irene Rivero-Calle, Pablo Rojo, Lorenza Romani, Prita Rughani, Luregn J. Schlapbach, Nina A. Schweintzger, Ching-Fen Shen, Artur Sulik, Maria Tsolia, Effua Usuf, Michiel van der Flier, Clementien Vermont, Ulrich von Both, Paul Wellman, Victoria J. Wright, Shunmay Yeung, Dace Zavadska, Aubrey J. Cunnington, Colin Fink, Jethro Herberg, Myrsini Kaforou, Shiranee Sriskandan, Michael Levin, Tom Parks

**Affiliations:** 1 Department of Infectious Disease, Imperial College London, London, United Kingdom; 2 Centre for Paediatrics and Child Health, Imperial College London, London, United Kingdom; 3 UKRI Centre for Doctoral Training in AI for Healthcare, Imperial College London, London, United Kingdom; 4 Micropathology Ltd, University of Warwick, Coventry, United Kingdom; 5 Centre for Bacterial Resistance Biology, Imperial College London, London, United Kingdom; 6 Antimicrobial Resistance & Healthcare-Associated Infection Division, UK Health Security Agency, London, United Kingdom; 7 NIHR Health Protection Research Unit in Healthcare Associated Infections and Antimicrobial Resistance, Imperial College London and UK Health Security Agency, London, United Kingdom; 8 Department of Pediatrics, Inselspital, Bern University Hospital, University of Bern, Bern, Switzerland; 9 Barts Health NHS Trust, London, United Kingdom; 10 Service de Pédiatrie-Urgences, AP-HP, Hôpital Louis-Mourier, Colombes, France; 11 Université Paris Cité, INSERM, IAME, Paris, France; 12 Department of Clinical Infection, Microbiology and Immunology, Veterinary and Ecological Sciences, University of Liverpool Institute of Infection, Liverpool, United Kingdom; 13 Department of Infectious Diseases, Alder Hey Children’s Hospital, Liverpool, United Kingdom; 14 Evelina London Children’s Hospital, Guy’s and St Thomas’ NHS Foundation Trust, London, United Kingdom; 15 Laboratory of Medical Immunology, Radboud Institute for Medical Innovation, Radboud UMC, Nijmegen, the Netherlands; 16 Radboud Community for Infectious Diseases, Radboud University Medical Center, Nijmegen, the Netherlands; 17 Translational and Clinical Research Institute, Newcastle University, Newcastle upon Tyne, United Kingdom; 18 Great North Children’s Hospital, Paediatric Immunology, Infectious Diseases & Allergy, Newcastle upon Tyne Hospitals NHS Foundation Trust, Newcastle upon Tyne, United Kingdom; 19 NIHR Newcastle Biomedical Research Centre Based at Newcastle upon Tyne Hospitals NHS Trust and Newcastle University, Newcastle upon Tyne, United Kingdom; 20 Brighton and Sussex Medical School, University of Sussex, East Sussex, United Kingdom; 21 Royal Alexandra Children’s Hospital, Brighton, United Kingdom; 22 University Childrens’ Hospital, University Medical Centre Ljubljana, Ljubljana, Slovenia; 23 Department of Pediatric Immunology, Rheumatology and Infectious Diseases, Amsterdam University Medical Center (Amsterdam UMC), Academic Medical Center (AMC), University of Amsterdam, Amsterdam, the Netherlands; 24 Sanquin Research Institute & Landsteiner Laboratory at the AMC, University of Amsterdam, Amsterdam, the Netherlands; 25 Translational Pediatrics and Infectious Diseases, Pediatrics Department, Hospital Clínico Universitario de Santiago, Santiago de Compostela, Spain; 26 GENVIP Research Group, Instituto de Investigación Sanitaria de Santiago, Universidad de Santiago de Compostela, Galicia, Spain; 27 CIBER of Respiratory Diseases (CIBERES), Instituto de Salud Carlos III, Madrid, Spain; 28 Department of General Paediatrics, Erasmus MC–Sophia Children’s Hospital, Rotterdam, the Netherlands; 29 Innovation & Partnerships, bioMérieux, Marcy l’Etoile, France; 30 Joint Research Unit Hospice Civils de Lyon–bioMérieux, Centre Hospitalier Lyon Sud, Pierre-Bénite, France; 31 EA 7426 Pathophysiology of Injury-Induced Immunosuppression, Lyon 1 University, Hospices Civils de Lyon–bioMérieux, Hôpital Edouard Herriot, Lyon, France; 32 Lewisham and Greenwich NHS Trust, London, United Kingdom; 33 Leeds Children’s Hospital, Leeds, United Kingdom; 34 NIHR Southampton Clinical Research Facility and Biomedical Research Centre, University Hospital Southampton NHS Foundation Trust, Southampton, United Kingdom; 35 Department of Paediatrics, University of Cambridge, Addenbrookes Hospital, Cambridge, United Kingdom; 36 Oxford Vaccine Group, Department of Paediatrics, University of Oxford, Oxford, United Kingdom; 37 NIHR Oxford Biomedical Research Centre, Oxford, United Kingdom; 38 Great Ormond Street Hospital for Children NHS Foundation Trust, London, United Kingdom; 39 Department of Infectious Diseases, University Medical Centre Ljubljana, Ljubljana, Slovenia; 40 Department of Paediatrics, Faculty of Medicine, University of Ljubljana, Ljubljana, Slovenia; 41 Servicio Madrileño de Salud (SERMAS), Pediatric Infectious Diseases Unit, Department of Pediatrics, Hospital Universitario 12 de Octubre, Madrid, Spain; 42 Department of Pediatrics, Faculty of Medicine, Universidad Complutense de Madrid, Madrid, Spain; 43 Infectious Disease Unit, Academic Department of Pediatrics, Bambino Gesù Children’s Hospital, IRCCS, Rome, Italy; 44 Department of Intensive Care and Neonatology, University Children’s Hospital Zurich, University of Zurich, Zurich, Switzerland; 45 Child Health Research Centre, University of Queensland, Brisbane, Queensland, Australia; 46 Department of Pediatrics and Adolescent Medicine, Division of General Pediatrics, Medical University of Graz, Graz, Austria; 47 Department of Pediatrics, National Cheng Kung University Hospital, College of Medicine, National Cheng Kung University, Tainan City, Taiwan; 48 Department of Pediatric Infectious Diseases, Medical University of Bialystok, Białystok, Poland; 49 National and Kapodistrian University of Athens, Second Department of Paediatrics, “P. and A. Kyriakou” Children’s Hospital, Athina, Greece; 50 Medical Research Council Unit, The Gambia at LSHTM, Fajara, The Gambia; 51 Wilhelmina Children’s Hospital, University Medical Center Utrecht, Utrecht, the Netherlands; 52 Department of Paediatric Infectious Diseases & Immunology, Erasmus MC–Sophia Children’s Hospital, Rotterdam, the Netherlands; 53 Division of Paediatric Infectious Diseases, Hauner Children’s Hospital, University Hospital, Ludwig Maximilians University, Munich, Germany; 54 German Center for Infection Research, Partner Site Munich, Munich, Germany; 55 Faculty of Infectious and Tropical Disease, London School of Hygiene and Tropical Medicine, London, United Kingdom; 56 Faculty of Public Health and Policy, London School of Hygiene and Tropical Medicine, London, United Kingdom; 57 Department of Paediatrics, St Mary’s Hospital Imperial College Hospital, London, United Kingdom; 58 Riga Stradins University, Riga, Latvia; 59 Children Clinical University Hospital, Riga, Latvia; 60 Centre for Human Genetics, University of Oxford, Oxford, United Kingdom

## Abstract

**Importance:**

The upsurge in invasive disease caused by *Streptococcus pyogenes* among children reported in several European countries during 2022 to 2023 has not been fully explained.

**Objective:**

To evaluate whether changes in the circulation of common respiratory pathogens associated with the introduction of nonpharmaceutical interventions (NPIs) during the COVID-19 pandemic were associated with acquisition of immunity to *S pyogenes* and common respiratory viruses.

**Design, Setting, and Participants:**

This cross-sectional study recruited children with suspected infection and afebrile control participants at hospitals in 10 European countries. Data were collected before (September 2016 to March 2020) and after (April 2020 to July 2023) the introduction of NPIs.

**Main Outcomes and Measures:**

Molecular detection of bacterial and viral pathogens on throat swabs and age-stratified total serum immunoglobin G (IgG) reactivity to *S pyogenes* cell wall extract from 2 strains, respiratory syncytial virus (RSV), 5 influenza viruses, 4 common cold coronaviruses, and SARS-CoV-2, measured by immunoassay.

**Results:**

Throat swabs from 1942 children aged 0 to 4 years were tested for respiratory pathogens (1449 recruited before introduction of NPIs [median (IQR) age, 19.7 (8.2-38.1) months; 798 (55.1%) male]; 493 recruited after [median (IQR) age, 20.7 (9.7-38.1) months; 269 (54.7%) male]). A decrease in detection of *S pyogenes,* RSV, common cold coronaviruses, and influenza viruses was observed between March 2020 to July 2021, corresponding to the maximal period of NPIs. Antibodies to *S pyogenes* were measured in 252 children recruited before NPIs and 200 thereafter. Antibodies to viral antigens were measured in 230 children before NPIs and 92 thereafter. Total IgG to *S pyogenes* and RSV was significantly lower in children aged 3 to 4 years recruited after NPI introduction compared with those recruited before (*S pyogenes emm1 *strain: after, 67 participants; median [IQR] 0.13 [0.44-0.44] relative units [RU]; before, 87 participants; median [IQR] 0.35 [0.10-0.65] RU; *P* = .007. RSV: after, 30 participants; median [IQR] 49.6 [31.1-120.7] mesoscale units [MU]/1000; before, 76 participants; median [IQR] 141.8 [78.1-423.1] MU/1000; *P* < .001). No such differences were observed for children aged 0 to 2 years or for individual influenza viruses or SARS-CoV-2.

**Conclusions and Relevance:**

In this cross-sectional study, there was a significant reduction in serum antibodies to *S pyogenes* and RSV in children aged 3 to 4 years after introduction of NPIs. Equivalent to approximately a 1-year delay in acquisition of immunity, these data suggest a putative biological basis for the 2022 to 2023 upsurge in severe *S pyogenes* infections in this age group.

## Key Points

**Question:**

Why did rates of life-threatening invasive *Streptococcus pyogenes* infections increase among children after the COVID-19 pandemic?

**Findings:**

In this cross-sectional study of 1942 children, 452 were tested for acquisition of antibody-mediated immunity to S pyogenes; the 67 children aged 3 to 4 years sampled after the introduction of nonpharmaceutical interventions early in the COVID-19 pandemic had significantly lower immunity compared with 87 similar children sampled before the pandemic. Acquisition of immunity to respiratory syncytial virus was similarly affected.

**Meaning:**

The findings of this study suggest that nonpharmaceutical interventions aimed at limiting SAR-CoV-2 infections during the COVID-19 pandemic may have had a profound impact on the development of immunity to *S pyogenes*, exposing the young children to this infection.

## Teaser

This cross-sectional study examines changes in immunity to *Streptococcus pyogenes *and other common respiratory pathogens among young children in 10 European countries after the introduction of nonpharmaceutical interventions during the COVID-19 pandemic.

## Introduction

A multicountry outbreak of severe invasive *Streptococcus pyogenes* (iGAS) infections was reported in the fourth quarter of 2022, with greatest impact on children younger than 10 years. This featured unusually severe presentations including rapidly progressive empyema and septic shock.^1,2,3^ In the UK, where infections normally peak between the first and second quarter,^4^ both iGAS disease and scarlet fever are notifiable. However, a sharp out-of-season increase in scarlet fever notifications in the fourth quarter was matched by a sharp increase in primary care consultations for streptococcal pharyngitis: both preceded a peak in iGAS notifications.^1^ For example, in the UK, surveillance of *S pyogenes* strains causing iGAS demonstrated an initial expansion of *emm12* strains in the second and third quarters of 2022, followed by a rapid increase in *emm1 S pyogenes* strains—mirrored in several European countries—in the fourth quarter of 2022, such that *emm1* accounted for approximately 70% of severe pediatric infections.^4,5,6,7^ Many of the children admitted to hospital with severe iGAS infection were reported to have coinfections with respiratory viruses, in particular respiratory syncytial virus (RSV).^4,8^ Moreover, coinfection with respiratory viruses was identified in more than half of the UK children who died in the community from iGAS infection during this period.^9^

Reduced exposure to common respiratory viruses and bacteria during the period of COVID-19–related restrictions has been postulated to result in loss of population immunity to common childhood pathogens.^10^ Scarlet fever is a disease that most commonly affects children in their first year at school.^11^ In the UK and other European countries, school attendance was reduced from March 2020, leading to reduced exposure to *S pyogenes*, rates of which fell markedly during the period March 2020 to March 2022.^2,12^ The term “immunity debt” was proposed by Cohen et al,^13^ who suggested that reduced circulation of common pathogens due to nonpharmaceutical interventions (NPIs) resulted in impaired community immunity to common infectious agents and thus to an increase in severe childhood illness when normal social interaction returned.^13^ The concept of immune debt (also called the immunity gap) has been widely debated in the scientific literature and popular press.^14,15^ However, although it is a plausible explanation for the increase in childhood illness following relaxation of pandemic social distancing measures, there is so far little biological data to support this hypothesis.^16^ Accordingly, using serum samples from children attending hospital as an indicator of community-level immunity in children, we investigated whether a reduction in acquisition of antibody-mediated immunity to common respiratory pathogens could be detected before and after introduction of NPIs.

## Methods

### Study Design

Our study comprised (1) a cross-sectional pathogen detection study covering September 2016 to July 2023 and (2) a cross-sectional study of antibody-mediated immunity to *S pyogenes* and common respiratory viruses before and after introduction of NPIs in March 2020. Both investigations focused on children attending hospitals in Europe recruited to 1 of 2 European Union–funded prospective, multicenter observational studies of febrile illness during childhood: PERFORM,^17^ recruiting from 19 hospitals in 9 European countries between September 2016 and March 2020, and DIAMONDS, recruiting from 20 hospitals in 6 European countries between March 2020 and July 2023.^18^

Ethical approval was obtained by each participating center from a Research Ethics Committee (REC) for both PERFORM and DIAMONDS (eTables 1 and 2 in Supplement 1). In the UK, Imperial College obtained approvals, as the coordinating site, from the UK Health Research Authority, after REC approvals for PERFORM and DIAMONDS. All children were recruited with informed written parental consent and assent from older children. We reported our study in accordance with the Strengthening the Reporting of Observational studies in Epidemiology (STROBE) reporting guidelines for cross-sectional studies.

Across both studies, children aged 0 to 18 years presenting with fever or suspected infection who were sufficiently unwell to require blood tests were recruited from emergency departments, pediatric inpatient wards, and pediatric intensive care units. Additionally, nonfebrile children who were undergoing blood sampling for unrelated reasons, including as outpatients, were recruited as control participants, providing they had not experienced fever or received a vaccination in the previous 3 weeks.^17^ As previously described, each child recruited to the study was assigned a final diagnostic category on the basis of the available clinical, laboratory, and imaging data by at least 2 experienced pediatricians using an algorithm modified from previous iterations^19^ to enhance detection of inflammatory disease (eFigure 1 in Supplement 1).

For our pathogen detection study, all children aged 0 to 4 years recruited to PERFORM and DIAMONDS (including control participants) were eligible if a throat swab had been taken during recruitment as part of our centralized molecular testing program, which was performed retrospectively as previously described to generate a comprehensive view of the pathogens present in the study population.^17^ For our antibody-mediated immunity study, we focused on a subset of children aged 0 to 4 years recruited to PERFORM and DIAMONDS who were as representative as possible of children in the wider population. To achieve this, we excluded children in the diagnostic categories representing definite or potentially life-threatening bacterial or viral infections, or definite inflammatory diagnoses (eFigure 1 in Supplement 1). Full inclusion and exclusion criteria are described in the eMethods in Supplement 1.

Ethnicity was self-reported by parents or guardians as part of the background information that was obtained from each participant. The DIAMONDS study used the following categories: Asian, Black, Middle Eastern, South American, White European, mixed, other, and not stated. The PERFORM study had minor changes in nomenclature as follows: Black was recorded as African/North African; White European as European; and South American as Meso/South American. For both studies, the other and mixed categories had no predefined subgroups and were recorded if selected by the parent or guardian. Free-text reporting was optional in both cases. Because DIAMONDS and PERFORM were established to develop novel diagnostic tests for childhood infectious and inflammatory disease, ethnicity information was collected to ensure candidate diagnostics performed equally well across ethnic groups.

### Data Sources

Deidentified clinical and demographic data were obtained from the PERFORM and DIAMONDS research databases derived from standardized case report forms. Children in definite bacterial, definite viral, or unknown bacterial or viral diagnostic categories, and 10% of all other categories, were cross-checked for inconsistencies, as previously described.^17^

For pathogen detection, throat swabs were tested using multiplex polymerase chain reaction (PCR) for respiratory pathogens including influenza A, influenza B, RSV, common cold coronaviruses, and *S pyogenes*. SARS-CoV-2 detection was performed on samples from 2020 on (eMethods in Supplement 1).

To measure antibody to *S pyogenes*, we adapted a previously reported enzyme-linked immunosorbent assay (ELISA) to measure absorbance for *S pyogenes* cell wall extracts^20,21^ of 2 representative strain types associated with scarlet fever: H305 (*emm1*/M1)^22^ and H690 (*emm12*/M12) (eMethods in Supplement 1).^23^ To measure antibodies to common respiratory viruses, sera from a subset of children diluted 1:5000 were further tested for total immunoglobin G (IgG) reactivity to viral analytes using the V-PLEX Respiratory Panel 1 and the V-PLEX COVID-19 Coronavirus Panel 3 kits (Meso Scale Discovery) according to the manufacturer’s protocol. Each sample was tested for reactivity to 15 analytes which, for this analysis, we focused on: hemagglutinin from 2 influenza A and 2 influenza B viruses; prefusion F-protein from RSV; spike proteins from 4 common cold coronaviruses; and 3 SARS-CoV-2 antigens (eMethods in Supplement 1). Finally, to contextualize our findings, we reviewed statutory notifications of iGAS and scarlet fever to the UK Health Security Agency (UKHSA) between January 2016 and December 2022 across England (eMethods in Supplement 1).

### Statistical Analysis

Data were inspected for missingness and outliers. All analyses were performed using R version 4.3.2 (R Project for Statistical Computing). Sample size was determined by sample availability at the time the experiments were conducted and the multiplex assay platform capacity, rather than a formal sample size calculation.

For the antibody-mediated immunity study, we divided children into 2 periods: those recruited before the first UK government–mandated lockdown on March 23, 2020 (termed before NPI), and those recruited after April 22, 2020 (termed after NPI); the 1-month gap reflected at least 1 half-life of natural IgG. To assess the association between age and reactivity before and after NPIs, age at sampling was categorized into 6 bands, beginning at 0 months, 6 months, 1 year, 2 years, 3 years, and 4 years. The first year was divided in half to allow appreciation of the decay of maternally derived antibody (eTable 7 in Supplement 2).

Unadjusted differences between time periods within age bands were tested using Wilcoxon rank-sum tests. Multivariable linear regression models with rank-normalized outcomes were used to adjust for potential confounders, with interactions assessed by likelihood ratio tests. Statistical significance was set at *P* < .05 for all analyses, and all tests were 2-sided. No adjustment for multiple testing was performed, as our primary hypothesis focused specifically on changes to *S pyogenes* immunity acquisition, and we did not consider it equally likely that all age groups or individual pathogens would be affected by timing related to the pandemic. Full details of our statistical methods are provided in the eMethods in Supplement 1.

## Results

Between September 2016 and July 2023, a total of 5484 children aged 0 to 4 years were recruited to the PERFORM and DIAMONDS studies at sites within Europe. Of these, 1942 (35.4%) had valid throat swab PCR results available, including 1449 prepandemic samples (median [IQR] age, 19.7 [8.2-38.1] months; 798 [55.1%] male; 89 [6.1%] Asian, 40 [2.8%] Black, and 1155 [797%] White European), and 493 after NPI introduction (median [IQR] age, 20.7 [9.7-38.1] months; 269 [54.7%] male; 86 [17.4%] Asian, 45 [9.1%] Black; and 272 [55.2%] White European) (Table). After exclusions, antibody titres to *S pyogenes emm1* and *emm12* were available from 452 children aged 0 to 4 years (153 [33.8%] with a valid throat swab PCR), while viral panel titres were available for 322 children (103 [32.0%] with a throat swab PCR) (eFigure 2 in Supplement 1).

**Table.** Baseline Characteristics of Participants

Characteristic	Participants, No. (%)							
	Pathogen detection				Antibody-mediated immunity			
	All^a^		Multiplex PCR		ELISA to *S pyogenes*		Multiplex	
	Before (n = 2802)^b^	After (n = 2682)^c^	Before (n = 1449)^b^	After (n = 493)^c^	Before (n = 252)^b^	After (n = 200)^c^	Before (n = 230)^b^	After (n = 92)^c^
Sex								
Male	1594 (56.9)	1511 (56.3)	798 (55.1)	269 (54.7)	147 (58.3)	127 (63.5)	136 (59.1)	60 (65.2)
Female	1204 (43.0)	1161 (43.3)	649 (44.8)	223 (45.2)	105 (41.7)	73 (36.5)	94 (40.9)	32 (34.8)
Missing sex	4 (0.1)	10 (0.3)	2 (0.1)	1 (0.2)	0	0	0	0
Age, median (IQR), mo^d^	20.5 (8.3-38.3)	22.0 (8.7-39.6)	19.7 (8.2-38.1)	20.7 (9.1-38.1)	26.8 (11.6-45.3)	25.3 (12.2-42.8)	26.3 (11.3-43.0)	25.7 (15.3-42.2)
Ethnicity^e^								
Asian	166 (5.9)	312 (11.6)	89 (6.1)	86 (17.4)	10 (4.0)	17 (8.5)	10 (4.3)	6 (6.5)
Black	106 (3.8)	254 (9.5)	40 (2.8)	45 (9.1)	7 (2.8)	20 (10.0)	6 (2.6)	6 (6.5)
Middle Eastern	58 (2.1)	29 (1.1)	20 (1.4)	8 (1.6)	<5	<5	<5	0
South American	<5	24 (0.9)	0	0	<5	<5	<5	<5
White European	2150 (76.7))	1511 (56.3)	1155 (79.7)	272 (55.2)	218 (86.5)	128 (64.0)	197 (85.7)	65 (70.7)
Mixed	67 (2.4)	68 (2.5)	37 (2.6)	17 (3.4)	<5	8 (4.0)	<5	5 (5.4)
Other	118 (4.2)	103 (3.8)	57 (4.0)	10 (2.0)	<5	<5	<5	0
Not stated	0	179 (6.7)	0	21 (4.3)	0	11 (5.5)	0	6 (6.5)
Missing ethnicity	136 (4.9)	201 (7.5)	51 (3.5)	34 (6.9)	7 (2.8)	9 (4.5)	7 (3.0)	3 (3.2)
Country^d^								
UK	955 (34.1)	1403 (52.3)	411 (28.4)	421 (85.4)	112 (44.4)	134 (67.0)	105 (45.6)	48 (52.2)
EU	1847 (65.9)	1279 (47.7)	1038 (71.6)	72 (14.6)	140 (55.5)	66 (33.0)	125 (54.3)	44 (47.8)
Diagnostic category^d^								
Definite bacterial	328 (11.7)	334 (12.5)	183 (12.6)	55 (11.2)	0	0	0	0
Probable bacterial	375 (13.4)	215 (8.0)	217 (15.0)	39 (7.9)	0	0	0	0
Bacterial syndrome	235 (8.4)	127 (4.7)	140 (9.7)	23 (4.7)	0	0	0	0
Unknown bacterial or viral	427 (15.2)	252 (9.4)	217 (15.0)	50 (10.1)	0	87 (43.5)	0	0
Viral syndrome	230 (8.2)	197 (7.3)	122 (8.4)	65 (13.2)	0	0	0	0
Probable viral	425 (15.2)	316 (11.8)	215 (14.8)	46 (9.3)	98 (38.9)	52 (26.0)	94 (40.9)	52 (56.5)
Definite viral	356 (12.7)	472 (17.6)	165 (11.4)	108 (22.0)	0	0	0	0
Inflammatory syndrome	56 (2.0)	197 (7.3)	24 (1.7)	55 (11.1)	0	0	0	0
Other phenotype^f^	151 (5.5)	383 (14.3)	71 (4.9)	47 (9.5)	0	20 (10.0)	0	0
Control or trivial	215 (7.7)	189 (7.0)	95 (6.6)	5 (1.0)	154 (61.1)	41 (20.5)	136 (59.1)	40 (43.5)

Abbreviations: ELISA, enzyme-linked immunosorbent assay; EU, European Union; PCR, polymerase chain reaction; *S pyogenes, Streptococcus pyogenes*.

^a^ All participants irrespective of availability of valid throat swab PCR results.

^b^ Before indicates before the introduction of nonpharmaceutical interventions during the COVID-19 pandemic, defined as before March 23, 2020.

^c^ After indicates after the lifting of nonpharmaceutical interventions during the COVID-19 pandemic, defined as after April 22, 2020.

^d^ No missingness in dataset for age, country of recruitment, or diagnostic category.

^e^ Ethnicity was self-reported by parents or guardians as part of the background information that was obtained from each participant. Other and mixed categories had no predefined subgroups and were recorded if selected by the parent or guardian. Free-text reporting was optional in both cases. The PERFORM study collected self-reported ethnicities with minor changes in nomenclature as follows: Black category was recorded as African/North African; White European as European; and South American as Meso/South American.

^f^ Comprising uncertain infection or inflammation, parasitic infections, and other unknown cause of illness.

In both the pathogen detection and antibody-mediated immunity subsets, characteristics of the participants were broadly similar before and after introduction of NPIs, although a higher proportion of samples afterwards came from Asian and Black children and were recruited in the UK. Additionally, unlike samples taken before NPIs, assessment of reactivity to *S pyogenes* after NPIs included 87 children (43.5%) from the unknown bacterial or viral infection category, 19 (9.5%) from uncertain infection or inflammation category, and 1 (0.5%) in the other cause of illness category.

**Figure 1.**
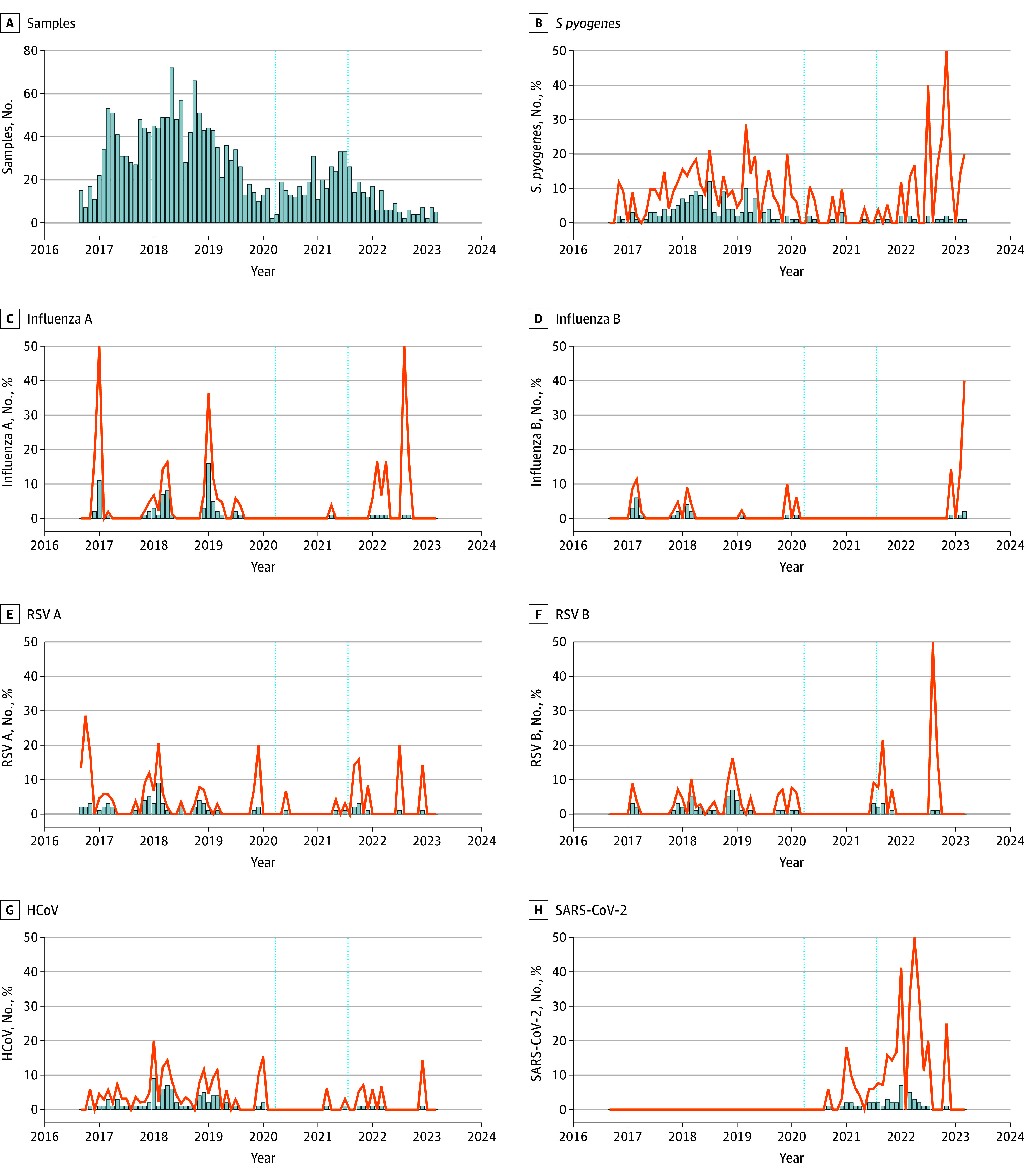
Timeseries Percentages of Monthly Throat Swab Polymerase Chain Reaction Detections for Respiratory Pathogens Detection of *Streptococcus pyogenes* and viral pathogens by polymerase chain reaction in children. Data are shown for children aged 0 to 4 years recruited to the PERFORM and DIAMONDS studies during September 2016 to August 2023. The monthly number of valid samples processed is shown in the first plot, followed by the monthly number of positive results for each pathogen, indicated using blue bars. The orange line indicates the monthly percentage of positive samples, plotted on the same scale. Dashed vertical lines indicate the dates of the introduction and lifting of restrictions in the UK, as described in the eMethods in Supplement 1. HCoV indicates common cold coronaviruses 229E, OC43, NL63, and HKU1 (combined); RSV, respiratory syncytial virus.

In the pathogen detection study, a marked decrease in the prevalence of detected pathogens, including RSV, common cold coronaviruses, and influenza viruses, was observed from March 2020 to July 2021, corresponding to the periods of social distancing and school closures (Figure 1). For example, RSV subtypes A and B were only detected in 3 of 16 months during the restrictions period, while human coronaviruses other than SARS-CoV-2 were only detected in 2 of 16 months. Detection of *S pyogenes* also declined during that period, with the pathogen detected in samples in 5 of 16 months during the restrictions period compared with 36 of 43 of months previously. Similar changes were seen in our wider cohort of children aged 0 to 18 years (eFigure 3 in Supplement 1).

**Figure 2.**
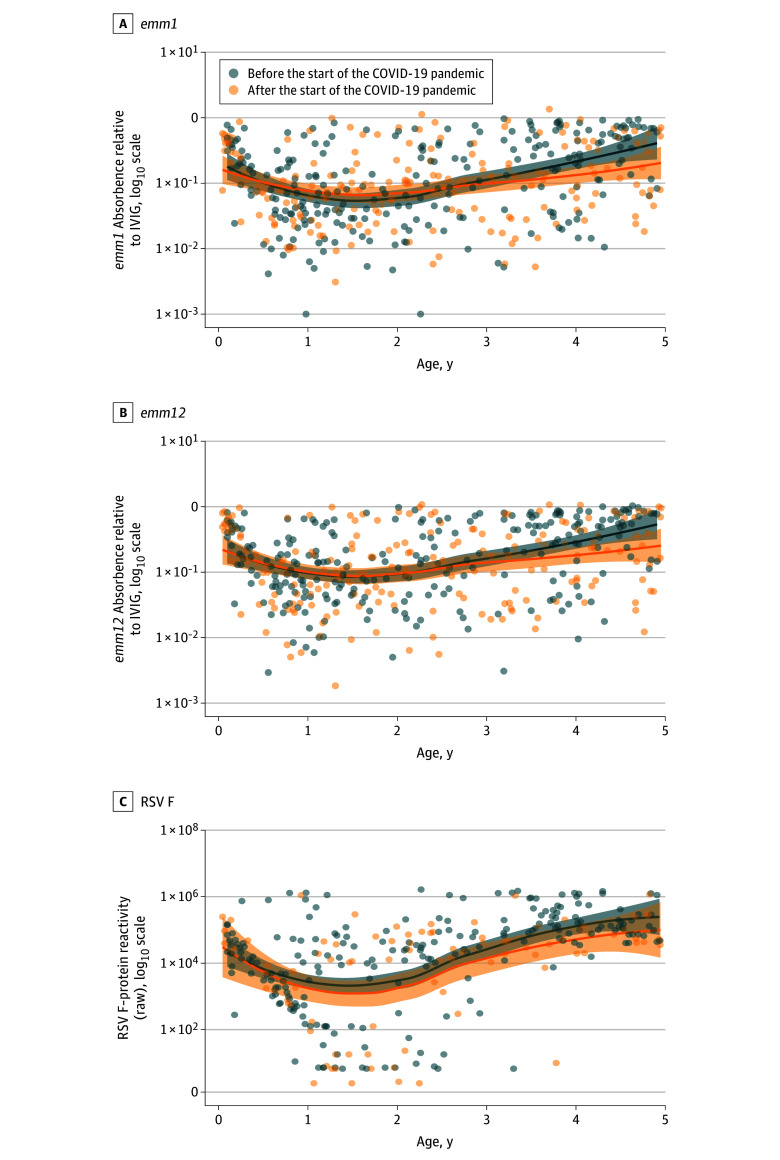
Reactivity to *Streptococcus pyogenes* and Respiratory Syncytial Virus (RSV) Antigens by Age Scatter plots show continuous age vs reactivity before nonpharmaceutical interventions for the COVID-19 pandemic (blue) and after (orange), with a trend line and 95% CI (shaded area) estimated by locally estimated scatterplot smoothing regression. IVIG indicates intravenous immunoglobin.

**Figure 3.**
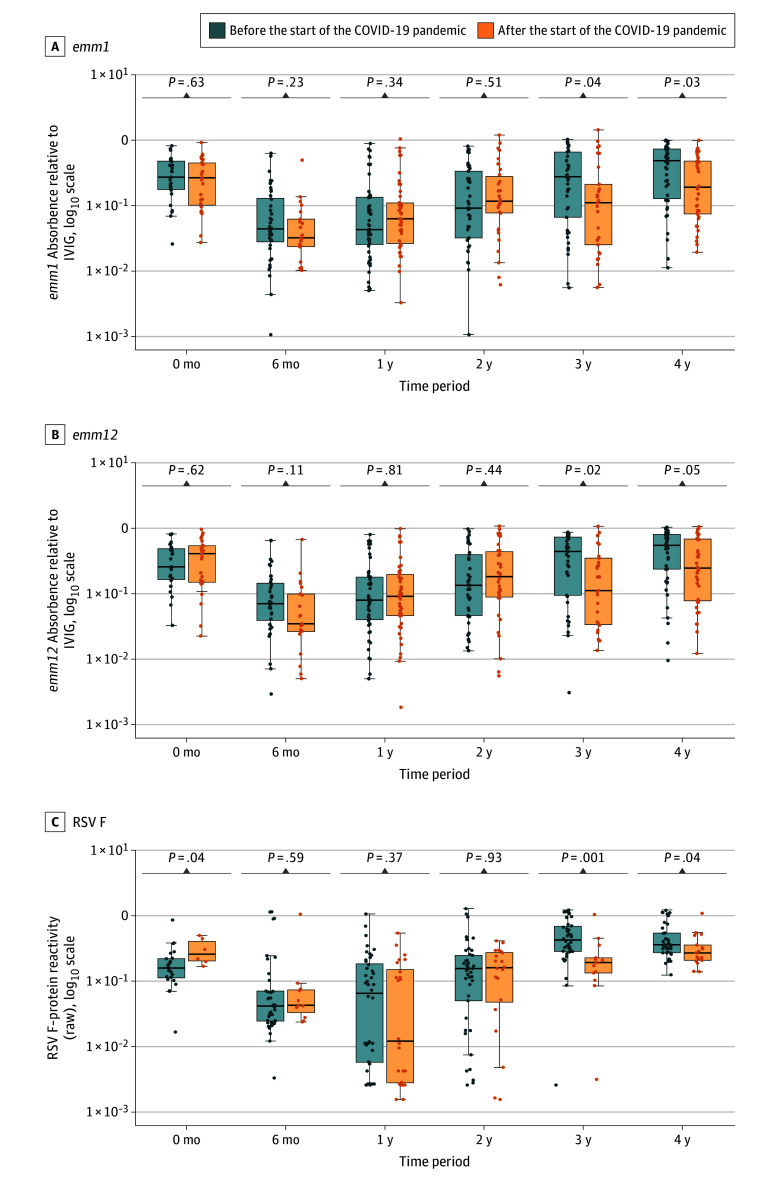
Reactivity to *Streptococcus pyogenes* and Respiratory Syncytial Virus (RSV) Antigens by Age Band Box plots show reactivity before nonpharmaceutical interventions for the COVID-19 pandemic (blue) and after (orange) with the difference between groups assessed by a Wilcoxon rank-sum test. Each box’s central line indicates the median; upper border of box, third quartile; lower border, first quartile; whiskers, 1.5 × IQR from box; and dots, individual data points. IVIG indicates intravenous immunoglobin.

Among 252 children sampled from before the pandemic for the antibody-mediated immunity study, reactivity to *S pyogenes* cell wall extract from *emm1* and *emm12*—which were highly correlated (*r* = 0.94; 95% CI, 0.92-0.95)—declined during the first year of life before increasing over the remaining years studied. However, this pattern was altered among the 200 children sampled after the introduction of NPIs (Figure 2A and B). Excluding 11 children (2.4%) in whom *S pyogenes* was detectable by PCR at the time of sampling (potentially reflecting acute infection), the median absorbance relative to intravenous immunoglobin among children aged 3 to 4 years was significantly lower after compared with before NPI (*S pyogenes emm1* strain: after, 67 participants; median [IQR], 0.13 [0.04-0.44] relative units [RU]; before, 87 participants; median [IQR] 0.35 [0.10-0.65] RU;*P* = .007; *emm12* strain: after, 67 participants, median [IQR], 0.21 [0.06-0.59] RU; before, 87 participants, median [IQR] 0.51 [0.18-0.76]; *P* = .005) (Figure 3A and B; eTable 3 in Supplement 1). In multivariable linear regression models, normalized absorbance for both *emm1* and *emm12* was similar among 3-year-old children recruited after NPIs to that of 2-year-old children either before or afterwards (eTables 4 and 5 in Supplement 1). Similarly, children older than 4 years recruited after the pandemic had similar normalized absorbance to 3-year-old children recruited before the pandemic. Further details of sensitivity and multivariable analyses are described in the eResults and eFigure 4 in Supplement 1.

A similar age-related acquisition of immunity was seen in a subset of 230 children recruited prior to the pandemic and 92 recruited thereafter in whom antibody reactivity to key antigens from common respiratory viruses were assessed using the multiplex assay. Among children aged 3 to 4 years, excluding 8 with detectable RSV at the time of sampling, reactivity to RSV was significantly lower among children recruited after introduction of NPIs (after, 30 participants; median [IQR] 49.6 [31.1-120.7] mesoscale units [MU]/1000; before, 76 participants; median [IQR] 141.8 [78.1-423.1] MU/1000; *P* < .001) (Figure 3C; eTable 3 in Supplement 1). The statistical association between RSV reactivity and age was broadly similar to that observed for *S pyogenes*, although the association with NPIs was only statistically significant in children aged 3 years (eTable 6 in Supplement 1).

**Figure 4.**
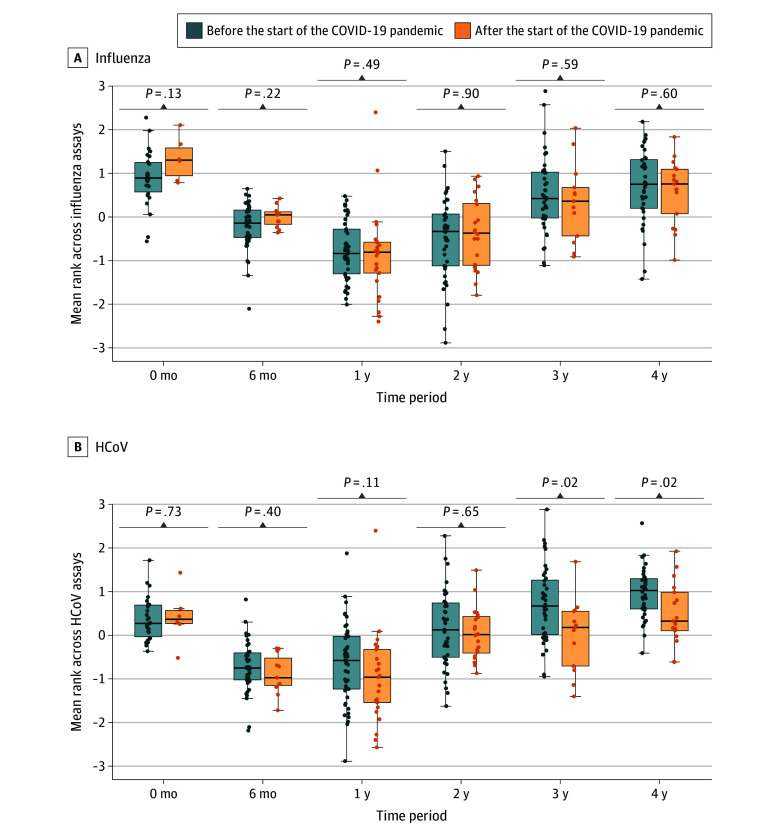
Aggregate Reactivity to Influenza Viruses and Common Cold Coronaviruses Box plots show reactivity before nonpharmaceutical interventions for the COVID-19 pandemic (blue) and after (orange), with the difference between groups assessed by a Wilcoxon rank-sum test. Each box’s central line indicates the median; upper border of box, third quartile; lower border, first quartile; whiskers, 1.5 × IQR from box; and dots, individual data points. HCoV indicates common cold coronaviruses 229E, OC43, NL63, and HKU1 (combined).

Correlation between reactivity to individual antigens was mostly explained by age but otherwise weak unless originating from the same or closely related viruses (eFigure 5 in Supplement 1). Although some other age-related changes were apparent, there were no consistent pandemic-related patterns of reactivity to individual influenza (eFigure 6 in Supplement 1) and a small reduction in 2 of 4 common cold coronavirus antigens (eFigure 7 in Supplement 1). Unlike the influenza viruses, reactivity to common cold coronaviruses in aggregate was also lower in children aged 3 to 4 years recruited after NPIs (Figure 4). In contrast to the other viruses, reactivity to SARS-CoV-2 antigens increased after NPIs, a pattern that was most apparent in samples obtained after July 2021 (eFigure 8 in Supplement 1). However, this increase was not apparent in all age groups (eFigure 9 in Supplement 1). Finally, in comparison with the prepandemic average, UKHSA notification data for England indicated a significantly greater increase in the incidence rate of iGAS infection but not scarlet fever during 2022 among children aged 3 to 4 years compared with those aged 0 to 2 years (eFigure 10 in Supplement 1).

## Discussion

We found a significant reduction in antibody concentrations against *S pyogenes* and RSV in children at 3 to 4 years of age attending hospitals in the UK and Europe in the first 3 years after the introduction of NPIs to combat the COVID-19 pandemic. Although we cannot infer causality from these data, this apparent reduction in antibody-mediated immunity coincides with the exact age group that experienced the greatest increase in life-threatening *S pyogenes* incidence after NPIs were removed in England, as confirmed by our analysis of UKHSA data. Moreover, the increase in iGAS cases occurred substantially earlier in the season than is typical, coinciding with a more seasonal but equally substantial increase in respiratory viruses, to which our data suggest children were also more susceptible. Ultimately these findings highlight the vulnerability of children to *S pyogenes* infection and the urgent need to develop a vaccine to prevent this disease.

The differences observed in our study appear restricted to preschool-aged children who are in the process of acquiring adaptive immunity to *S pyogenes.* Our multivariable analyses suggested that, on average, the antibody-mediated immunity of children recruited after NPI introduction was a year behind that of those recruited before the pandemic. Thus, given the vital importance of antibody in preventing *S pyogenes* infection, scaled to the population, this delayed acquisition of immunity could have contributed to the increase of iGAS infections observed during 2022 to 2023. While the dataset pertaining to viral antigens was smaller, acquisition of immunity to RSV also appeared delayed, although the reduction appeared limited to children aged 3 years. To some extent this difference may reflect an artifact of measuring reactivity to a single RSV antigen rather than the entire *S pyogenes* cell wall, but further work would be required to investigate whether reactivity to single *S pyogenes* antigens is acquired at a different rate. Our data also suggest NPIs may have altered acquisition of immunity to common cold coronaviruses but not influenza viruses, at least as assessed in this multiplex assay. This result may reflect the complex range of factors influencing population-level immunity to influenza viruses, including influenza vaccinations.

Similar findings relating to *S pyogenes* and RSV antibody levels have been reported by 1 study focusing on adult blood donors sampled in New Zealand.^24^ While limited to adults, who experienced a more marginal increase in iGAS during 2022 to 2023,^25,26^ that study benefited from repeated measures from the same individuals and assessed individual *S pyogenes* antigens rather than our cell wall–extract approach. Thus, the concordance with our results in children is notable and supportive of our conclusions. Others have reported waning antibody-mediated immunity to RSV in women of child-bearing age and infants in Canada^27^ and across the age spectrum in the Netherlands.^28^ Another group in China observed similar age-specific changes in RSV antibodies among children aged 0 to 4 years, finding a pronounced decline in children aged 3 to 4 years during the pandemic.^29^

### Limitations

This study has limitations. While the results of our pathogen detection study resemble the findings of others,^30^ these data were not available for the entire cohort, and a high proportion of samples after March 2020 were obtained in the UK. Keeping in mind the limited extent to which it is possible to sample healthy children, most children included in our antibody-mediated immunity study are representative of those aged 0 to 4 years in the wider population. Nonetheless, we acknowledge that we included children from a wider range of diagnostic categories in our antibody-mediated immunity study recruited during and after the pandemic. Additionally, our study is limited by samples from a single time point, by a relatively small sample size, and to children aged 0 to 4 years, and we had fewer total samples and a greater proportion obtained in the UK after the pandemic, introducing the possibility of selection bias. However, adjustment for these factors in our multivariable analyses did not alter our results, although our estimates of the differences in antibody levels before and after the pandemic may be imprecise. We recognize that additional unmeasured confounders may impact our results, and we are unable to determine the impact of NPIs in individual countries. It is also unclear how changes in health-seeking behavior or daycare attendance during this period might have altered our results. Further work would be needed to investigate whether the reduction in *S pyogenes* binding persisted further into childhood. Moreover, we would have ideally extended the analysis of reactivity to viral antigens to a larger number of samples but that was beyond the scope of the current project. In future work we will investigate the extent to which the patterns observed for *S pyogenes* cell wall antigen would be recapitulated in binding to single antigens. Additionally, our work was limited to measurement of total IgG to a limited range of antigens, and we have not yet studied other isoforms or subclasses of antibody or more unbiased approaches to characterizing antibody and other aspects of adaptive immunity to *S pyogenes*.

## Conclusions

In this cross-sectional study, we observed a putative immunological explanation for the upsurge in severe *S pyogenes* infections affecting younger children and the wider population in 2022 to 2023. Most importantly, beyond their implications for use of NPIs in response to future pandemics, these findings strengthen the case for *S pyogenes* vaccine development to protect vulnerable groups in the wider population.^31^

## Notes

Supplement 1.
eMethods.

eResults.
**eTable 1.** Participating Clinical Recruitment Sites for PERFORM **eTable 2.** Participating Clinical Recruitment Sites for DIAMONDS **eTable 3.** Raw Location Estimates and Unadjusted Wilcoxon Rank-Sum Tests for Differences in Absorbance Relative to IVIG of S pyogenes emm1 and emm12 and Reactivity to RSV F-Protein by Age Band **eTable 4.** Multiple Linear Regression of Inverse Rank Normalized Absorbance Relative to IVIG for S pyogenes emm1 Cell Wall Extract **eTable 5.** Multiple Linear Regression of Inverse Rank Normalized Absorbance Relative to IVIG for S pyogenes emm12 Cell Wall Extract **eTable 6.** Multiple Linear Regression of Inverse Rank Normalized Reactivity to RSV F-Protein **eFigure 1.** Diagnostic Algorithm Used for Classification **eFigure 2.** Selection of Samples for Antibody-Mediated Immunity Study **eFigure 3.** Detection of Pathogens at Age 0 to 17 Years **eFigure 4.** Reactivity to S pyogenes by Age Band in Sensitivity Analyses **eFigure 5.** Correlation of Reactivity Between Antigens **eFigure 6.** Reactivity to Influenza Virus Haemagglutinins by Age Band **eFigure 7.** Reactivity to Common Cold Coronavirus Spike Proteins by Age Band **eFigure 8.** Reactivity to SARS-CoV-2 Antigens and RSV F-Protein Across All Ages **eFigure 9.** Reactivity to SARS-CoV-2 Antigens by Age Band **eFigure 10.** Changes in S pyogenes Disease Incidence by Age
eReferences.

Supplement 2. **eTable 7.** Subset Data Release

Supplement 3.
Nonauthor Collaborators

Supplement 4.
Data Sharing Statement
