# Supplementary material for: Immunity to Streptococcus pyogenes and Common Respiratory Viruses at Age 0 to 4 Years After COVID-19 Restrictions
Source: JAMA Netw Open. 2025 Oct 15;8(10):e2537808. doi: 10.1001/jamanetworkopen.2025.37808 (PMC12529189; doi:10.1001/jamanetworkopen.2025.37808)
Supplement: Supplement 1. — eMethods. eResults. eTable 1. Participating Clinical Recruitment Sites for PERFORM eTable 2. Participating Clinical Recruitment Sites for DIAMONDS eTable 3. Raw Location Estimates and Unadjusted Wilcoxon Rank-Sum Tests for Differences in Absorbance Relative to IVIG of S pyogenes emm1 and emm12 and Reactivity to RSV F-Protein by Age Band eTable 4. Multiple Linear Regression of Inverse Rank Normalized Absorbance Relative to IVIG for S pyogenes emm1 Cell Wall Extract eTable 5. Multiple Linear Regression of Inverse Rank Normalized Absorbance Relative to IVIG for S pyogenes emm12 Cell Wall Extract eTable 6. Multiple Linear Regression of Inverse Rank Normalized Reactivity to RSV F-Protein eFigure 1. Diagnostic Algorithm Used for Classification eFigure 2. Selection of Samples for Antibody-Mediated Immunity Study eFigure 3. Detection of Pathogens at Age 0 to 17 Years eFigure 4. Reactivity to S pyogenes by Age Band in Sensitivity Analyses eFigure 5. Correlation of Reactivity Between Antigens eFigure 6. Reactivity to Influenza Virus Haemagglutinins by Age Band eFigure 7. Reactivity to Common Cold Coronavirus Spike Proteins by Age Band eFigure 8. Reactivity to SARS-CoV-2 Antigens and RSV F-Protein Across All Ages eFigure 9. Reactivity to SARS-CoV-2 Antigens by Age Band eFigure 10. Changes in S pyogenes Disease Incidence by Age eReferences. [file jamanetwopen-e2537808-s001.pdf]

Supplemental Online Content

Dokal K, Channon-Wells S, Davis C, et al. Immunity to *Streptococcus pyogenes* and common respiratory viruses at age 0 to 4 years after COVID-19 restrictions. *JAMA Netw Open*. 2025;8(10):e2537808. doi:10.1001/jamanetworkopen.2025.37808

eMethods.
eResults.
eTable 1. Participating Clinical Recruitment Sites for PERFORM
eTable 2. Participating Clinical Recruitment Sites for DIAMONDS
eTable 3. Raw Location Estimates and Unadjusted Wilcoxon Rank-Sum Tests for Differences in Absorbance Relative to IVIG of S pyogenes emm1 and emm12 and Reactivity to RSV F-Protein by Age Band
eTable 4. Multiple Linear Regression of Inverse Rank Normalized Absorbance Relative to IVIG for S pyogenes emm1 Cell Wall Extract
eTable 5. Multiple Linear Regression of Inverse Rank Normalized Absorbance Relative to IVIG for S pyogenes emm12 Cell Wall Extract
eTable 6. Multiple Linear Regression of Inverse Rank Normalized Reactivity to RSV F-Protein
eFigure 1. Diagnostic Algorithm Used for Classification
eFigure 2. Selection of Samples for Antibody-Mediated Immunity Study
eFigure 3. Detection of Pathogens at Age 0 to 17 Years
eFigure 4. Reactivity to S pyogenes by Age Band in Sensitivity Analyses
eFigure 5. Correlation of Reactivity Between Antigens
eFigure 6. Reactivity to Influenza Virus Haemagglutinins by Age Band
eFigure 7. Reactivity to Common Cold Coronavirus Spike Proteins by Age Band
eFigure 8. Reactivity to SARS-CoV-2 Antigens and RSV F-Protein Across All Ages
eFigure 9. Reactivity to SARS-CoV-2 Antigens by Age Band
eFigure 10. Changes in S pyogenes Disease Incidence by Age
eReferences.

This supplemental material has been provided by the authors to give readers additional information about their work.

© 2025 Dokal K et al. *JAMA Network Open*.

eMethods

1. Study design – Selection of participants

For our antibody-mediated immunity study, we aimed to obtain a cross-sectional sample that was as representative as possible of children in the wider population, with the caveat that, due to the smaller number of samples available from March 2020 onwards, it was necessary to include children from a wider range of diagnostic categories. Thus, for the period before introduction of NPIs, we selected children from three diagnostic categories (controls, probable viral infection and trivial illness), whereas, for children recruited during or after the pandemic, we included all children with an aliquot of serum available at the time of the experiments from six diagnostic categories (controls, probable viral infection, trivial illness, uncertain infection or inflammation, unknown bacterial of viral infection, other cause of illness). To increase parity between the groups, we selected children for the pre-pandemic period with an aliquot of serum available if they resembled a child included during or after the pandemic in terms of at least one of age band, season at time of sampling, and site of recruitment. Subsequently, children were excluded if: they had been assessed as immunocompromised; they had received intravenous immunoglobulin (IVIG) prior to sampling; a sample from a previous timepoint had been included; they were recruited during the first month of non-pharmaceutical interventions (NPIs) in the UK (23rd March to 22nd April 2020); or a subsequent review of the case report form revealed age greater than 5 years or lead to a change to their diagnostic category (eFigure 2). Finally, due to limited capacity on the Meso Scale Discovery (MSD) platform, we limited assessment of antibody-mediated immunity to common respiratory viruses to children in the three overlapping diagnostic categories (i.e. controls, probable viral infection and trivial illness) and randomly removed a further 23 children selected during “shortlisting ” to achieve approximately equally sized age categories among the pre-pandemic samples.

2. Data sources – Pathogen detection

For our pathogen detection study, dry flocked throat swabs stored in eNAT™ media were tested retrospectively as part of our centralised molecular testing programme on the MAGPIX® system by Luminex Diasorin, using the NxTAG™ Respiratory Pathogen Panel (RPP).[1] This gave results for 16 viral and three bacterial targets from which, for this analysis, we focused on influenza A (subtypes H1 and H3), influenza B, RSV (subtypes A/B), and common cold coronaviruses (HKU1, NL63, 229E and OC43). Additionally, we used qualitative PCR to detect S. pyogenes targeting the gene for DNase B using a previously described method developed at Micropathology Ltd.[1] Finally, for samples collected from 2020 onwards,

© 2025 Dokal K et al. *JAMA Network Open*.

we used a reverse transcription quantitative PCR, also developed at Micropathology Ltd, to detect SARS-CoV-2 targeting the N gene as previously described.[2]

3. Data sources – *S. pyogenes* cell wall ELISA

Following optimisation, colonies for H305 (*emm1*/M1) and H690 (*emm12*/M12) were streaked onto Columbia blood agar, before they were inoculated into 50ml Todd-Hewitt broth and incubated overnight at 37C with 5% CO2. Overnight cultures were centrifuged at 12,000 x g and bacterial pellets were resuspended in 1 ml cell wall extraction buffer (10mM Tris-HCL; 30% w/v raffinose [Sigma-Aldrich®; Cat. R0250]; 0.1kU/ml Mutanolysin [Sigma-Aldrich®; Cat. M9901]; 1mg/ml lysozyme [Sigma-Aldrich®; Cat. L6876]; and 10ul protease inhibitor cocktail III [VWR, cat. no. 535140]). Following incubation for three hours at 37°C, samples were centrifuged at 15,000 x g for 10 minutes. The supernatant (cell wall extract) was collection, clarified by filtration (0.2μm) and dialysed overnight into PBS solution at 4°C using a 20kDa MWCO cassette (Thermo Scientific™; Cat. 88528). Cell wall extracts were then concentrated using a centrifugal filter unit (Thermo Scientific™; Cat. G9023). Protein concentration for each of the cell wall extracts were measured using a Pierce™ BCA Protein assay kit (Thermo™; Cat 23227) and followed as per the protocol.

Following optimisation in serum samples from healthy adults with non-group A streptococcus infections (without immunosuppression or prior treatment with intravenous immunoglobulin), 1 ug of cell wall extract (H305 and H690) was coated onto the wells of high binding 96-well plates overnight at 4°C. Wash buffer (0.05% [v/v] Tween-20 in phosphate buffered saline [PBS]) was used to wash the plates before 50 μl blocking buffer (5% bovine serum albumin, 0.1% Tween-20, 0.1% normal goat serum [Sigma-Aldrich®; Cat. G9023] in PBS) was added to each well and incubated for 1 hour at room temperature. After a further washing step, 50μl of serum samples (diluted to 1:1000 in blocking buffer) was added in duplicate for 1 hour at room temperature. After washing the plate three times, 50μl of Fc region specific, Horseradish peroxidase-conjugated goat anti-human antibody (Sigma-Aldrich®; Cat. A0170) diluted 1:60,000 in blocking buffer was added and incubated for 1 hour at room temperature. Wells were washed three times before adding 50μl of 3,3′,5,5′-Tetramethylbenzidine (Sigma-Aldrich®; Cat. T0565) for 25 minutes, protected from light. Next, 25μl of 1M sulphuric acid was added and absorbance was immediately measured at 450nm (Thermo Scientific™ Multiskan™ FC Microplate Photometer; Cat. 51119000) and readings were subtracted from a reference wavelength of 570nm. After subtraction of the blank readings, duplicate absorbance readings were averaged. Absorbance readings were recorded relative to 1:1000 IVIG (Privigen, CSL Behring) run in duplicate for each respective plate. Finally, to assess for non-

© 2025 Dokal K et al. *JAMA Network Open*.

specific binding, adult samples were also tested with blocking solution containing normal rabbit serum, alongside normal rabbit serum incubated instead of patient samples.

For final analysis, two individuals with M1 reactivity below the limit of detection were assigned arbitrary values of 0.001.

4. Data sources – Multiplex viral antigens assay

Each sample was tested for reactivity to 15 analytes from which, for this analysis, we focused on: hemagglutinin from two influenza A viruses (A/Michigan/45/2015[H1N1] and A/Hong Kong/4801/2014[H3N2]) and two influenza B viruses (B/Phuket/3073/2013 and B/Brisbane/60/2008); prefusion F-protein from RSV; spike proteins from four common cold coronaviruses (HKU1, NL63, 229E and OC43); and nucleocapsid, spike and spike receptor binding domain (RBD) from SARS-CoV-2.

5. Data sources – UKHSA Notification Data

We reviewed statutory notifications of invasive *Streptococcus pyogenes* (iGAS) infections and scarlet fever to the UK Health Security Agency (UKHSA) from across England for children aged 0-4 years made between January 2016 December 2022. More specifically, iGAS infection (sterile-site isolates) reports were extracted from routine and reference laboratory national databases, then merged and grouped into 14-day non-repeating episodes.[3] Scarlet fever notifications between were extracted from the UKHSA notifications of infectious diseases database.[4]

6. Statistical analysis – Pathogen detection study

For the pathogen detection study, data were transformed to a times series using the R package *tsibble*.[5] Monthly totals and the proportion of positive samples were calculated with both the two influenza A subtypes and the four common cold coronaviruses each pooled together.

7. Statistical analysis – Unadjusted estimates of reactivity

Estimates of difference in the location parameter for unadjusted absorbance relative to IVIG for *S. pyogenes* and reactivity to RSV F-protein in mesoscale-units by age band were calculated using a Wilcoxon rank-sum test, implemented in R using the wilcox.test function. We report estimates of difference in location and 95% confidence intervals calculated using the Hodges-Lehmann estimator.[6]

© 2025 Dokal K et al. *JAMA Network Open*.

8. Statistical analysis – Time periods relative to pandemic

Our main analysis used a division into two time periods as described in the main text. As a sensitivity analysis, we divided the time after 22nd April 2020 into the periods during which the majority of NPIs were in place (termed 'During') up to 19th July 2021 and after this date when they were lifted (termed 'Easing'). This date was chosen because it was when the UK Government moved to 'step 4' of its COVID-19 roadmap, including lifting of all remaining limits on social contact.[7] Notably, as these dates all reflected general trends in use of NPIs across Europe, time periods were applied across the entire dataset, rather than using different dates for each of the regions that recruited to the study. Kruskal-Wallis tests were used to test the statistical significance of differences between reactivity between these three time periods for individual age bands.

9. Statistical analysis – Multiplex viral assay data for antibody study

We combined reactivity to multiple antigens for groups of related viruses (e.g. influenza viruses) by ranking raw MSD values for each individual assay, and then taking the mean rank across all assays for each patient.

10. Statistical analysis – Multivariable linear regression for antibody study

Multivariable linear regression analyses were performed to test the relationship between age reactivity to *S. pyogenes* and RSV F-protein with adjustment for potential confounders. Reactivity to *S. pyogenes* (measured as absorbance for *S. pyogenes* cell wall extracts relative to IVIG) and RSV F-protein (measure using MSD V-PLEX panels) were transformed to approximate a normal distribution using a rank-based inverse normal transform, implemented with the RankNorm function from the *RNOmni* package.[8]

Age was categorized into six bands starting at 0 months, 6 months, 1 year, 2 years, 3 years and 4 years. The 6-11 months group was used as the reference category as this captures the nadir of circulating IgG in early childhood, when the majority of maternally acquired IgG has been catabolised, before significant endogenous IgG production.[9] Models were adjusted for covariates selected *a priori* to minimize confounding: diagnostic category (control/trivial illness versus unwell child), recruitment site (UK versus Europe), and biological sex.

To assess the impact of NPIs on age-related antibody acquisition, we included interaction terms between age bands and timing of sample collection (before versus after March 2020).

© 2025 Dokal K et al. *JAMA Network Open*.

The statistical significance of interactions was evaluated by comparing the log-likelihood of models with and without the interaction terms (likelihood ratio tests). This approach enabled us to determine whether the pattern of age-dependent antibody acquisition differed between children recruited before and after the introduction of pandemic-related NPIs

Finally, we assessed correlation between age-adjusted reactivity to distinct antigens by calculating the Pearson's correlation coefficient (r) for each relationship using the *corrplot* package,[10] using the residuals from a linear regression model for the rank-normalised reactivity with parameters for each of age bands to adjust for age.

11. Statistical analysis – UKHSA notification data

To understand changes in the incidence of iGAS and scarlet fever among children aged 3-4 years, relative to those aged 0-2 years, we calculated yearly incidence rates for each disease for each age band based on the total number of notifications divided by the UK Office for National Statistics population estimate for England for that each bracket. We then examined the change in incidence of each disease in age band relative to the respective pre-pandemic average for 2016 to 2019.

12. Statistical analysis – Sensitivity analyses

For our pathogen detection study, we repeated our analysis extending inclusion to all children between the ages of 0-18 years of age recruited to both studies.

To determine the robustness of the results of our antibody-mediated immunity study, our analysis of absorbance relative to IVIG of *S. pyogenes* was repeated under the following conditions: inclusion of the 11 children with detection of *S. pyogenes* by PCR at the time of the serum sample; exclusion of three children with detection of SARS-CoV-2 by PCR; and dividing the timing of recruitment into three periods, with time after 22nd April 2020 divided into the periods during which the majority of NPIs were in place (termed 'During') up to 19th July 2021 and after this date when they were lifted (termed 'Easing'). Finally, we tested for differences in absorbance relative to IVIG for *S. pyogenes* and reactivity to each of the viral antigens relative to the batches in which each of the assays were performed using Kruskal-Wallis tests.

13. Statistical analysis – Additional analysis packages

Plots were generated using the *ggplot2* and *ggpubr* packages in R.[11,12]

© 2025 Dokal K et al. *JAMA Network Open*.

eResults

1. Sensitivity analyses for *S. pyogenes*

We observed differences in absorbance for *S. pyogenes* cell wall extract from *emm1* and *emm12* in children aged 3-4 years relative to the introduction of NPIs across three sensitivity analyses. These included analyses with inclusion of the 11 children with detection of *S. pyogenes* by PCR at the time of the serum sample (eFigure 4A), exclusion of the three with detection of SARS-CoV-2 (eFigure 4B), and dividing the timing of recruitment into three periods (See also: 12. Statistical analysis – Time periods relative to pandemic; eFigure 4C). There was also no evidence of statistically significant differences between the batches in which the assays were performed.

2. Multivariable linear regression for *S. pyogenes*

Differences in normalised absorbance for *S. pyogenes* cell wall extract from *emm1* and *emm12* in children aged 3-4 years relative to the introduction of NPIs were also apparent after adjustment for potential confounders using multivariable linear regression. Specifically, after adjusting for diagnostic category, recruitment site and biological sex, children aged 3-4 years had an increment in absorbance for *S. pyogenes emm1* that was 0.36 normalised units lower than children of the same age recruited before March 2020 (beta -0.36, 95% CI, -0.65 to -0.06, p=0.02; eTable 4). We observed a similar pattern for *S. pyogenes emm12* with an increment that was 0.32 normalised units lower (beta -0.32, 95% CI, -0.62 to -0.03, p=0.03; eTable 5).

© 2025 Dokal K et al. *JAMA Network Open*.

eTable 1. Participating Clinical Recruitment Sites for PERFORM

**Country**	**Participating site**	**Ethical approval number**
United Kingdom	• St Mary's Hospital, Imperial College Healthcare NHS Trust, London<br>• Alder Hey Children's Hospital, Liverpool<br>• Great North Children's Hospital, Newcastle upon Tyne<br>• John Radcliffe Hospital, Oxford<br>• Royal Alexandra Children's Hospital, Brighton	16/LO/1684
Austria	• Medizinische Universität Graz, Graz	28-518 ex 15/16
Germany	• Dr. von Hauner Children's Hospital, Ludwig- Maximilians-University, Munich	699-16
Greece	• P. and A. Kyriakou Children's Hospital, Athens	415/13.06.16
Latvia	• Children's Clinical University Hospital, Riga	1/16-07-14
Netherlands	• Sophia's Children's Hospital, Rotterdam<br>• Academic University Medical Center, Amsterdam<br>• Radboud University Medical Center, Nijmegen	NL58103.091.16
Slovenia	• University Medical Centre Ljubljana	0120-483/2016-3
Spain	• Hospital Clínico Universitario de Santiago de Compostela	2016/331
Switzerland	• University Children's Hospital, Universität Bern, Bern	2016-01835

© 2025 Dokal K et al. *JAMA Network Open*.

eTable 2. Participating Clinical Recruitment Sites for DIAMONDS

Country	Participating site	Ethical approval number
United Kingdom	• St Mary's Hospital, Imperial College Healthcare NHS Trust, London<br>• Alder Hey Children's Hospital, Liverpool<br>• Great North Children's Hospital, Newcastle upon Tyne<br>• Southampton Children's Hospital, Southampton<br>• Evelina London Children's Healthcare, London<br>• Royal Alexandra Children's Hospital, Brighton<br>• John Radcliffe Hospital, Oxford<br>• The Leeds Teaching Hospitals NHS Foundation Trust, Leeds<br>• Leicester Children's Hospital, Leicester<br>• Addenbrookes Hospital, Cambridge<br>• University Hospital Lewisham, London<br>• Royal London Hospital and Newham Hospital, Barts Health NHS Trust, London<br>• Great Ormond Street Hospital, London	20/HRA/1714
Austria	• Medizinische Universität Graz, Graz	32-401 Ex 19/20
Germany	• Dr. von Hauner Children's Hospital, Ludwig- Maximilians-University, Munich	20-0568
Greece	• P. and A. Kyriakou Children's Hospital, Athens	9707/21.05.2020

© 2025 Dokal K et al. *JAMA Network Open*.

eTable 2 (continued): Participating Clinical Recruitment Sites for DIAMONDS

Country	Participating site	Ethical approval number
Italy	• Università degli Studi di Milano Statale, Milan<br>• Ospedale Pediatrico Bambino Gesù, Rome	0018932-U 27/05/202 & 848196
Latvia	• Children's Clinical University Hospital, Riga	Nr. 01-29.1/2736
Netherlands	• University Medical Center Utrecht, Utrecht<br>• Erasmus Medical Center., Rotterdam<br>• Amsterdam University Medical Center, Amsterdam	20-774/M (NL75190.041.20)
Slovenia	• University Medical Centre Ljubljana	0120-271/2020/3
Spain	• Hospital Clínico Universitario de Santiago de Compostela<br>• Servicio Madrileño de Salud, Madrid	2020/219 & 20/234
Switzerland	• University Children's Hospital, Universität Bern, Bern<br>• University Children's Hospital Zürich, Zürich	2020-01556

© 2025 Dokal K et al. *JAMA Network Open*.

eTable 3. Raw Location Estimates and Unadjusted Wilcoxon Rank-Sum Tests for Differences in Absorbance Relative to IVIG of *S pyogenes emm1* and *emm12* and Reactivity to RSV F-Protein by Age Band

Reactivity	Age	Timing Relative to the Pandemic			
		Median		Wilcoxon rank-sum test	
		Before March 2020	After March 2020	Pseudomedian Difference* (95% CI)	P-value
**Absorbance for *emm1* relative to IVIG, (relative units)**	0 months	0.26	0.25	-0.03 (-0.13 to 0.08)	0.63
	6 months	0.04	0.03	-0.01 (-0.04 to 0.01)	0.23
	1 year	0.04	0.06	0.01 (-0.01 to 0.03)	0.34
	2 years	0.09	0.11	0.02 (-0.04 to 0.07)	0.51
	3 years	0.26	0.10	-0.09 (-0.24 to -0.00)	0.04
	4 years	0.46	0.18	-0.12 (-0.27 to -0.01)	0.03
**Absorbance for *emm12* relative to IVIG, (relative units)**	0 months	0.26	0.41	0.03 (-0.09 to 0.20)	0.62
	6 months	0.07	0.03	-0.03 (-0.06 to 0.00)	0.11
	1 year	0.08	0.09	0.00 (-0.03 to 0.04)	0.81
	2 years	0.13	0.18	0.03 (-0.04 to 0.11)	0.44
	3 years	0.44	0.11	-0.17 (-0.33 to -0.01)	0.02
	4 years	0.55	0.25	-0.14 (-0.33 to -0.00)	0.05
**Reactivity to RSV F-protein, (mesoscale units/ 1000)**	0 months	25.4	70.4	35.9 (5.0 to 169.5)	0.04
	6 months	1.7	1.8	0.25 (-2.0 to 1.8)	0.59
	1 year	4.4	0.15	-0.02 (-7.7 to 0.12)	0.37
	2 years	24.2	25.8	0.05 (-15.7 to 22.7)	0.93
	3 years	181.2	37.0	-113.1 (-300.7 to -44.6)	0.001
	4 years	129.4	72.5	-52.9 (-113.6 to -1.1)	0.04

*Pseudomedian difference denotes the Hodges-Lehmann estimator

© 2025 Dokal K et al. *JAMA Network Open*.

eTable 4. Multiple Linear Regression of Inverse Rank Normalized Absorbance Relative to IVIG for *S pyogenes emm1* Cell Wall Extract (n=441).

**Age Band**	**Timing Relative to the Pandemic**					
	**Before March 2020**		**After March 2020**			
	**beta (95% CI)**	**p-value**	**beta (95% CI)**	**p-value**	**beta (95% CI) for Timing Relative to Pandemic within strata of Age**	**p-value**
**0 months**	1.07 (0.73 to 1.41)	<0.001	1.22 (0.83 to 1.62)	<0.001	0.15 (-0.08 to 0.38)	0.20
**6 months**	0	-	0.15 (-0.08 to 0.38)	0.20		
**1 year**	0.21 (-0.09- to 0.51)	0.20	0.36 (0.01 to 0.71)	0.05		
**2 years**	0.58 (0.27 to 0.89)	<0.001	0.73 0.37 to 1.10)	<0.001		
**3 years**	1.00 (0.66 to 1.35)	<0.001	0.64 (0.28 to 1.01)	<0.001	-0.36 (-0.65 to -0.06)	0.02
**4 years**	1.33 (0.99 to 1.67)	<0.001	0.97 (0.63 to 1.32)	<0.001		

Measure of interaction between After March 2020 and Age 3 or 4 years, beta -0.51 (95% CI -0.87 to -0.15), P=0.006
Likelihood ratio test for model with vs without interaction between After March 2020 and Age 3 or 4 years, P=0.005
Estimates are adjusted for Biological sex, Recruitment site (Europe vs UK) and Diagnostic category (unwell child vs control/trivial). The ‘unwell child’ category included probable viral, unknown bacterial or viral, uncertain infection or inflammation or other unknown cause of illness.
CI, confidence intervals.

© 2025 Dokal K et al. *JAMA Network Open*.

eTable 5. Multiple Linear Regression of Inverse Rank Normalized Absorbance Relative to IVIG for *S pyogenes emm12* Cell Wall Extract (n=441)

**Age Band**	**Timing Relative to the Pandemic**					
	**Before March 2020**		**After March 2020**			
	**beta (95% CI)**	**p-value**	**beta (95% CI)**	**p-value**	**beta (95% CI) for Timing Relative to Pandemic within strata of Age**	**p-value**
**0 months**	1.07 (0.73 to 1.40)	<0.001	1.23 (0.84 to 1.62)	<0.001	0.17 (-0.06 to 0.39)	0.15
**6 months**	0	-	0.17 (-0.06 to 0.39)	0.15		
**1 year**	0.24 (-0.06 to 0.53)	0.12	0.40 (0.05 to 0.75)	0.02		
**2 years**	0.62 (0.31 to 0.92)	<0.001	0.78 (0.42 to 1.15)	<0.001		
**3 years**	0.97 (0.63 to 1.31)	<0.001	0.65 (0.29 to 1.01)	<0.001	-0.32 (-0.62 to -0.03)	0.03
**4 years**	1.38 (1.05 to 1.72)	<0.001	1.06 (0.72 to 1.41)	<0.001		

Measure of interaction between After March 2020 and Age 3 or 4 years, beta -0.49 (95% CI -0.85 to -0.13), P=0.008
Likelihood ratio test for model with vs without interaction between After March 2020 and Age 3 or 4 years, P=0.007
Estimates are adjusted for Biological sex, Recruitment site (Europe vs UK) and Diagnostic category (unwell child vs control/trivial). The ‘unwell child’ category included probable viral, unknown bacterial or viral, uncertain infection or inflammation or other unknown cause of illness.
CI, confidence intervals.

© 2025 Dokal K et al. *JAMA Network Open*.

eTable 6. Multiple Linear Regression of Inverse Rank Normalized Reactivity to RSV F-Protein (n=314)

**Age Band**	**Timing Relative to the Pandemic**					
	**Before March 2020**		**After March 2020**			
	**beta (95% CI)**	**p-value**	**beta (95% CI)**	**p-value**	**beta (95% CI) for Timing Relative to Pandemic within strata of Age**	**p-value**
**0 months**	0.49 (0.12 to 0.87)	0.01	0.32 (-0.12 to 0.75)	0.20	-0.18 (-0.40 to 0.04)	0.11
**6 months**	0	-	-0.18 (-0.40 to 0.04)	0.11		
**1 year**	-0.23 (-0.54 to 0.08)	0.14	-0.41 (-0.76 to -0.05)	0.03		
**2 years**	0.28 (-0.03 to 0.59)	0.07	0.10 (-0.26 to 0.47)	0.60		
**3 years**	1.30 (0.95 to 1.65)	<0.001	0.52 (0.02 to 1.01)	0.04	-0.78 (-1.29 to -0.27)	0.003
**4 years**	1.23 (0.91 to 1.54)	<0.001	1.05 (0.68 to 1.42)	<0.001	-0.18 (-0.40 to 0.04)	0.11

Measure of interaction between After March 2020 and Age 3 years, beta -0.61 (95% CI -1.16 to -0.55), P=0.03
Likelihood ratio test for model with vs without interaction between After March 2020 and Age 3 years, P=0.03
Likelihood ratio test for supplementary model with vs without additional interaction term between After March 2020 and Age 4 years, P=0.57
Estimates are adjusted for Biological sex, Recruitment site (Europe vs UK) and Diagnostic category (unwell child vs control/trivial). The 'unwell child' category included probable viral, unknown bacterial or viral, uncertain infection or inflammation or other unknown cause of illness.
CI, confidence intervals

© 2025 Dokal K et al. *JAMA Network Open*.

eFigure 1. Diagnostic Algorithm Used for Classification

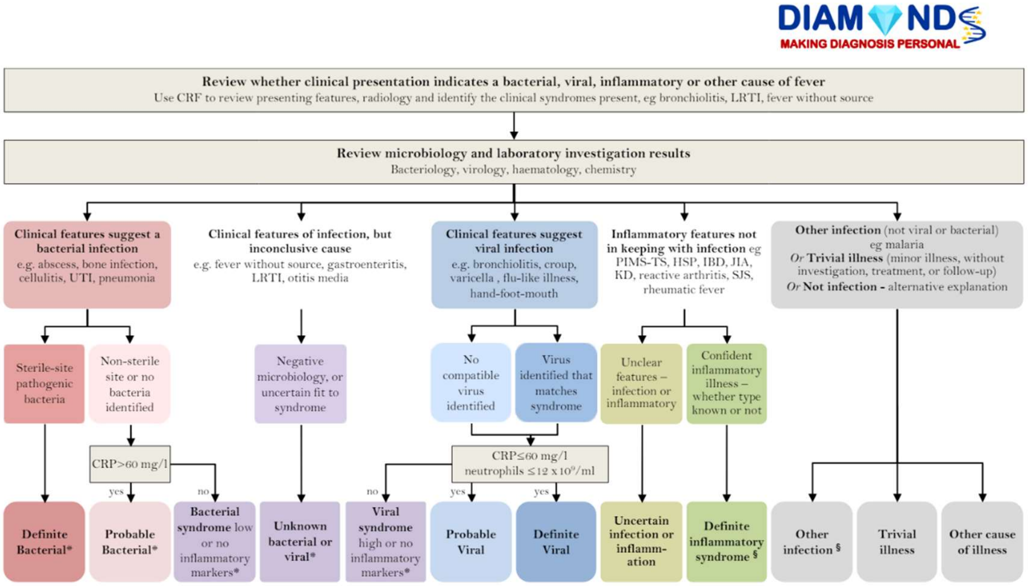

* Detection of virus does not exclude attribution of these infection phenotypes
§ Note that two phenotypes can be assigned to a patient if one is a non-viral/non-bacterial infection, or an inflammatory phenotype

eFigure 2. Selection of Samples for Antibody-Mediated Immunity Study

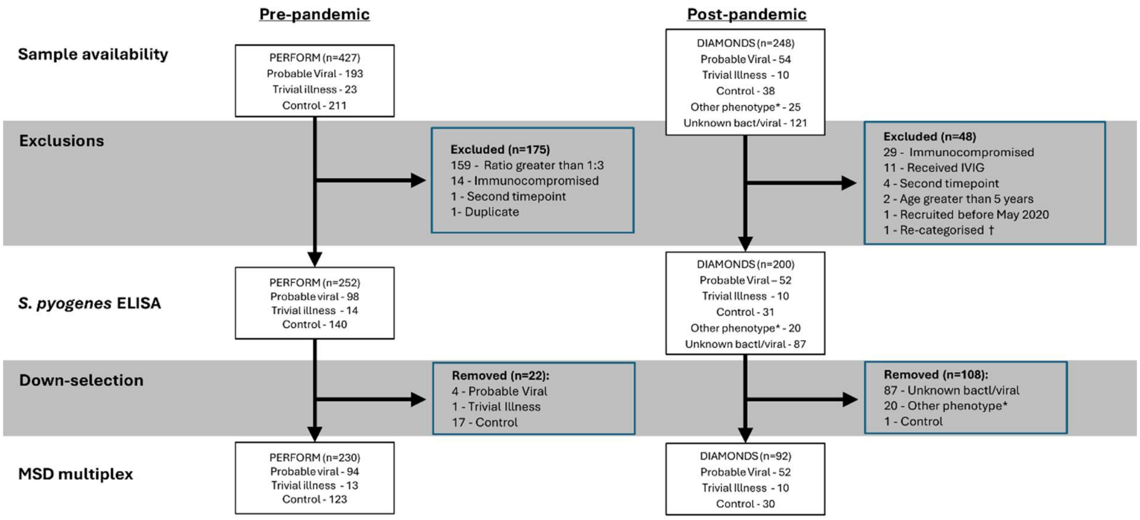

© 2025 Dokal K et al. *JAMA Network Open*.

eFigure 3. Detection of Pathogens at Age 0 to 17 Years

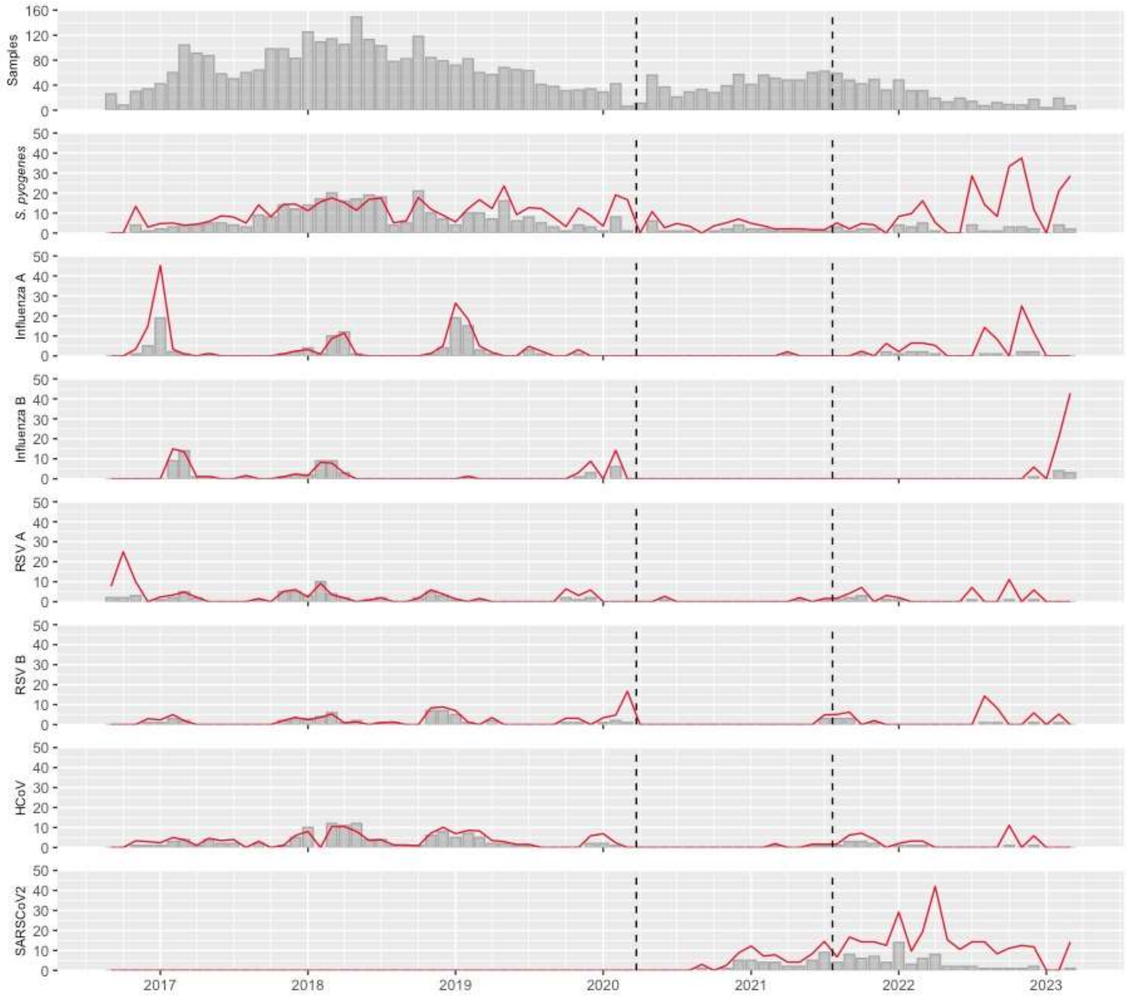

Detection of *S. pyogenes* and viral pathogens by PCR among children aged 0-17 years (n=4,130) recruited to the PERFORM and DIAMONDS studies during September 2016 to August 2023. The monthly number of valid samples processed is shown in the first plot followed by the monthly number of positives for each pathogen indicated using grey bars. The red line indicates the monthly percentage of positive samples. Dashed vertical lines indicate the dates of introduction and cessation of restrictions in the UK. RSV, respiratory syncytial virus; HCoV, common cold coronaviruses 229, OC43, NL63 and HKU1 (combined); SARSCoV2, Severe acute respiratory syndrome coronavirus 2.

© 2025 Dokal K et al. *JAMA Network Open*.

eFigure 4. Reactivity to *S pyogenes* by Age Band in Sensitivity Analyses

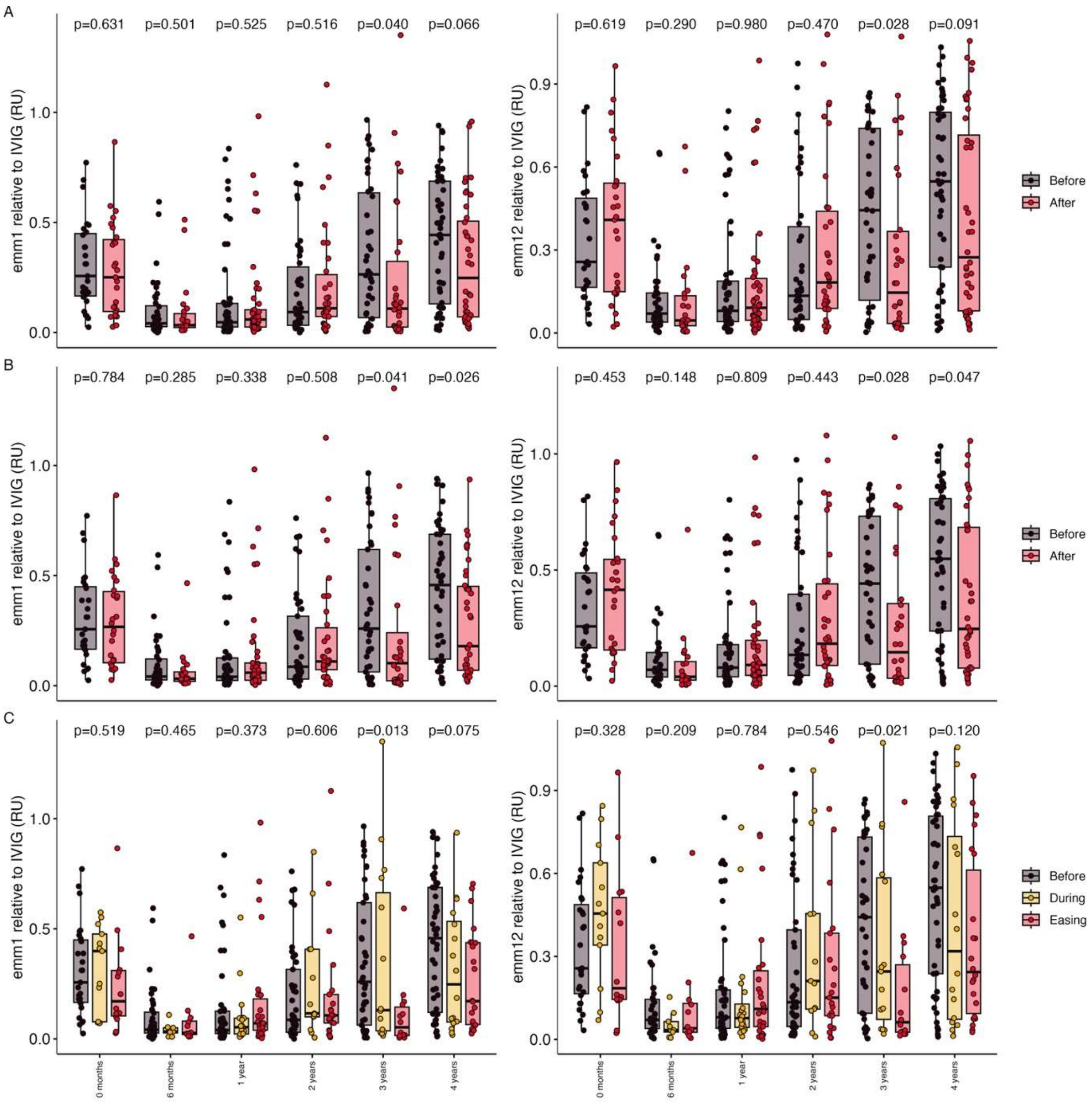

Boxplots for *emm1* (left) and *emm12* (right) show absorbance relative to IVIG: **A**. Irrespective of detection of *S. pyogenes* by PCR at the time of sampling; **B**. Excluding individuals with detection of SARS-CoV-2; and **C**. Dividing timing of recruitment into three periods. Differences between before (black) and after (red) March 2020 were assessed by a Wilcoxon rank-sum test, while differences before March 2020 (black), April 2020 to July 2021 (yellow) or after July 2021 (red) were assessed using a Kruskal-Wallis test.

© 2025 Dokal K et al. *JAMA Network Open*.

eFigure 5. Correlation of Reactivity Between Antigens

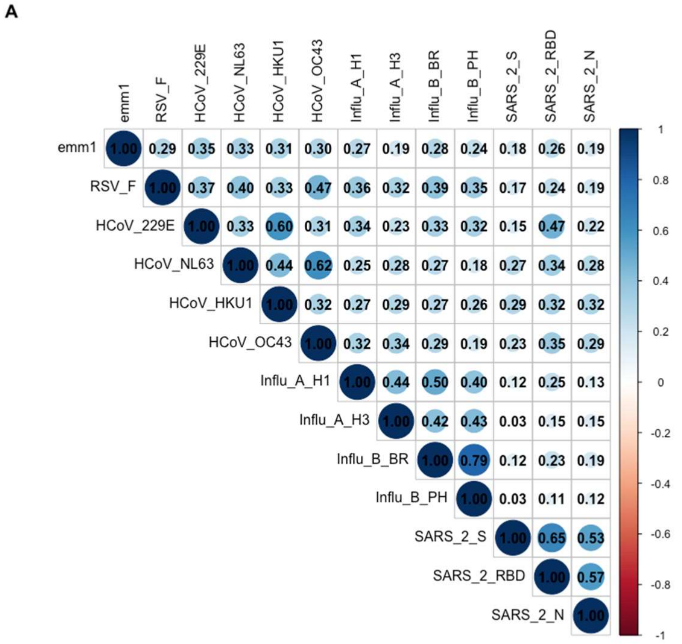

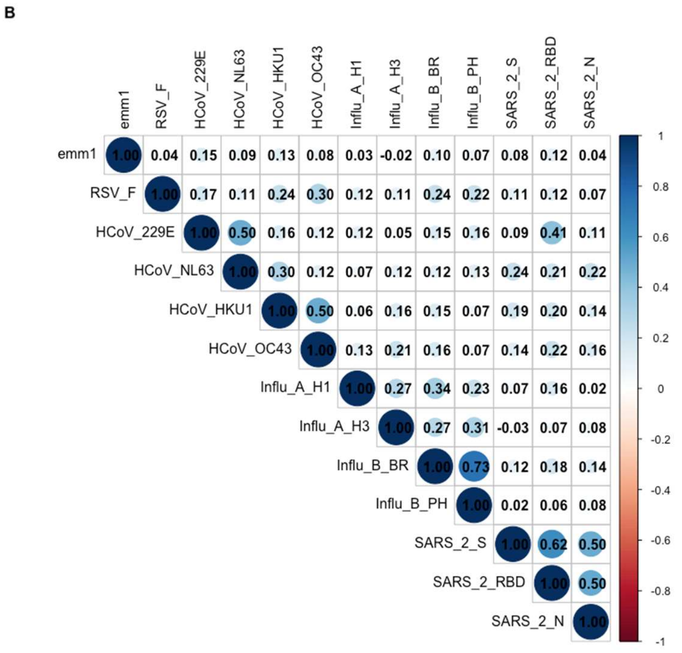

Pearson's correlation coefficient (*r*) for the relationship between reactivity to *S. pyogenes* and the 12 viral antigens in 230 samples used in the MSD assay. **A.** Unadjusted rank-normalised reactivity; **B.** Age-adjusted rank-normalised reactivity as the residuals from a linear regression model for rank-normalised reactivity with parameters for each of the age bands.

© 2025 Dokal K et al. *JAMA Network Open*.

eFigure 6. Reactivity to Influenza Virus Haemagglutinins by Age Band

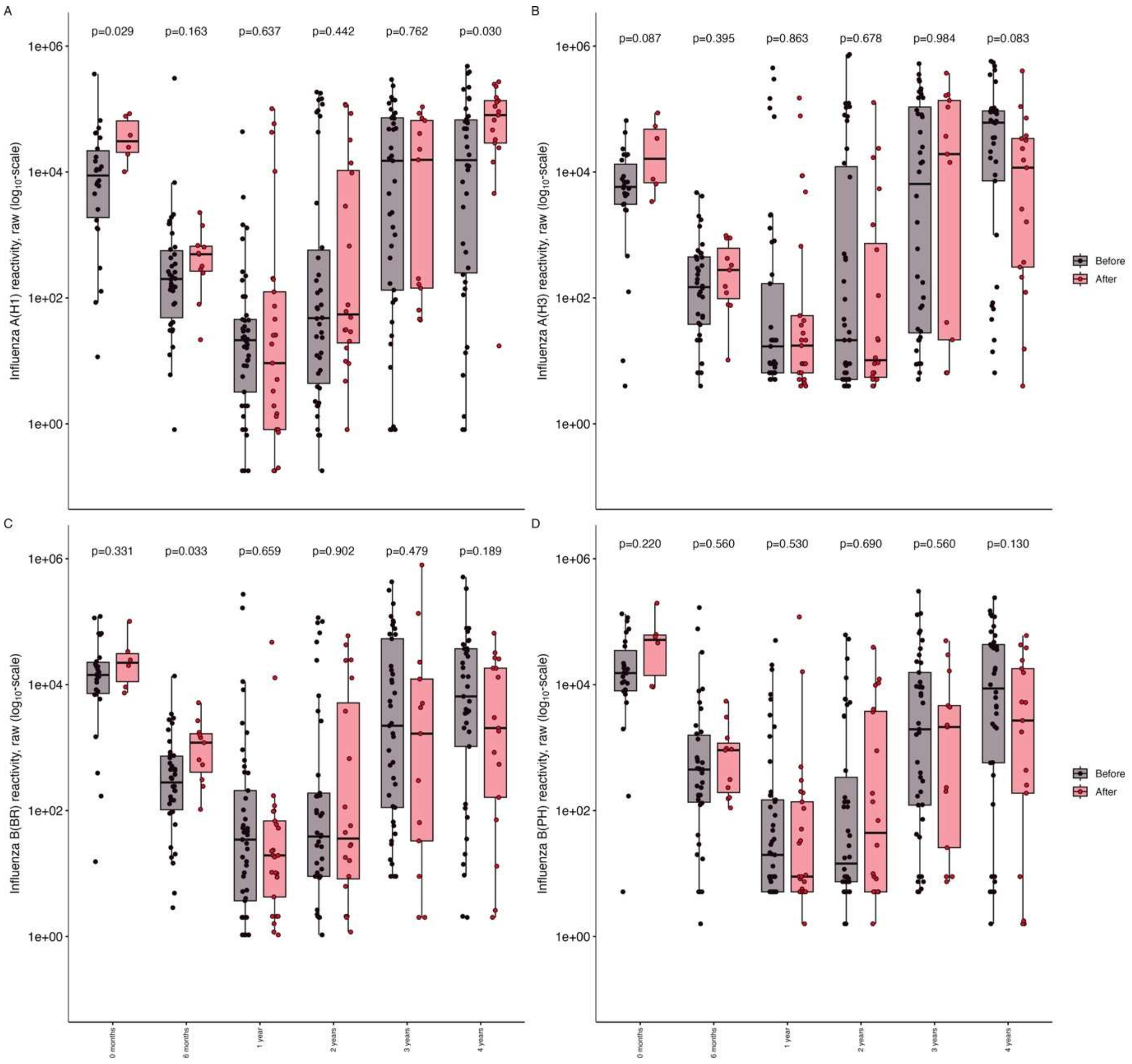

Box plots show reactivity before (black) and after (red) March 2020 with the difference between groups assessed by a Wilcoxon rank-sum test. **A**. Influenza A (H1N1); **B.** Influenza A (H3N2); **C**. Influenza B (Brisbane); and **D**. Influenza B (Phuket).

© 2025 Dokal K et al. *JAMA Network Open*.

eFigure 7. Reactivity to Common Cold Coronavirus Spike Proteins by Age Band

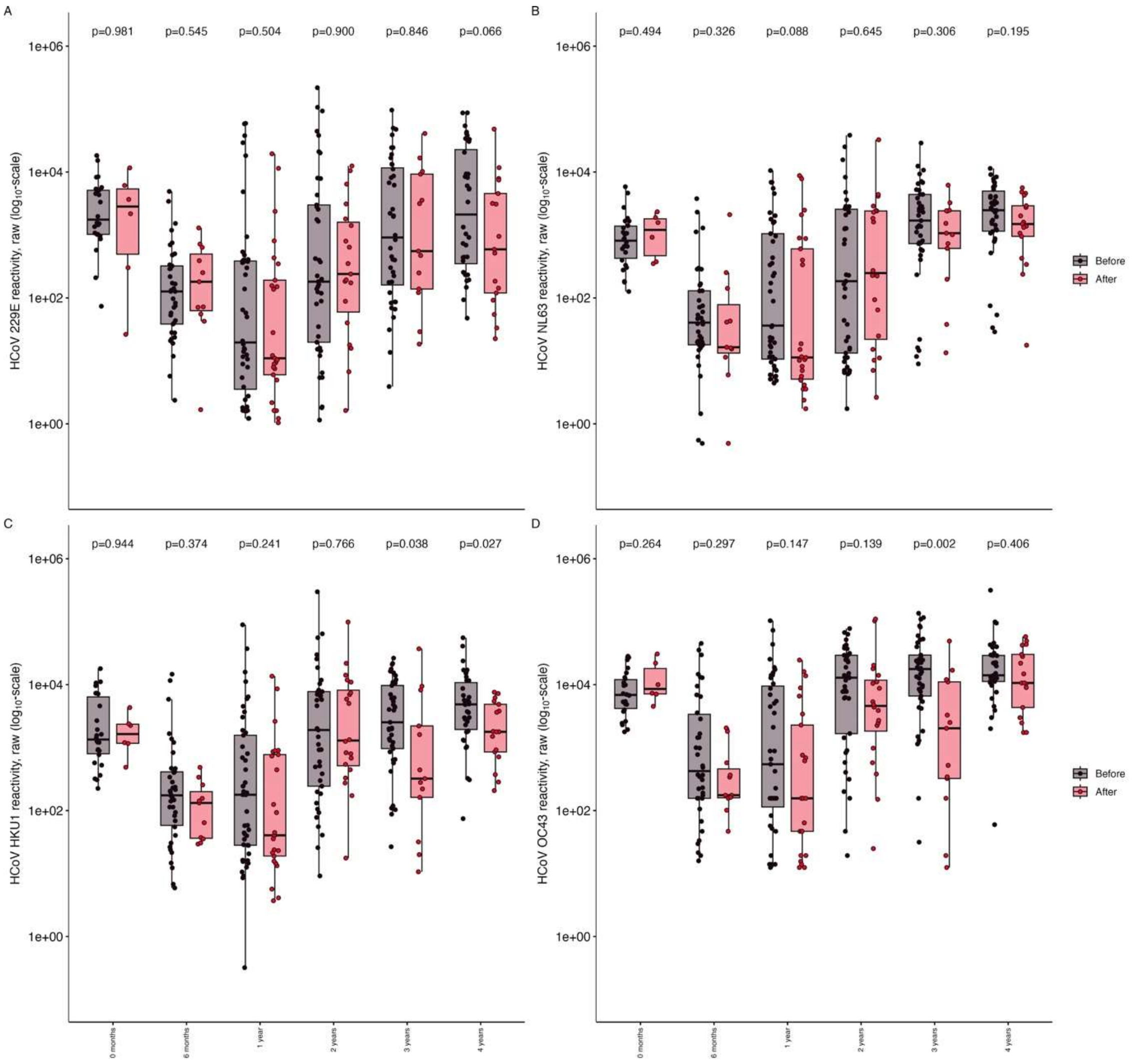

Box plots show reactivity before (black) and after (red) March 2020 with the difference between groups assessed by a Wilcoxon rank-sum test. **A**. 229E; **B.** HKU1; **C**. OC43; and **D**. NL63.

© 2025 Dokal K et al. *JAMA Network Open*.

eFigure 8. Reactivity to SARS-CoV-2 Antigens and RSV F-Protein Across All Ages

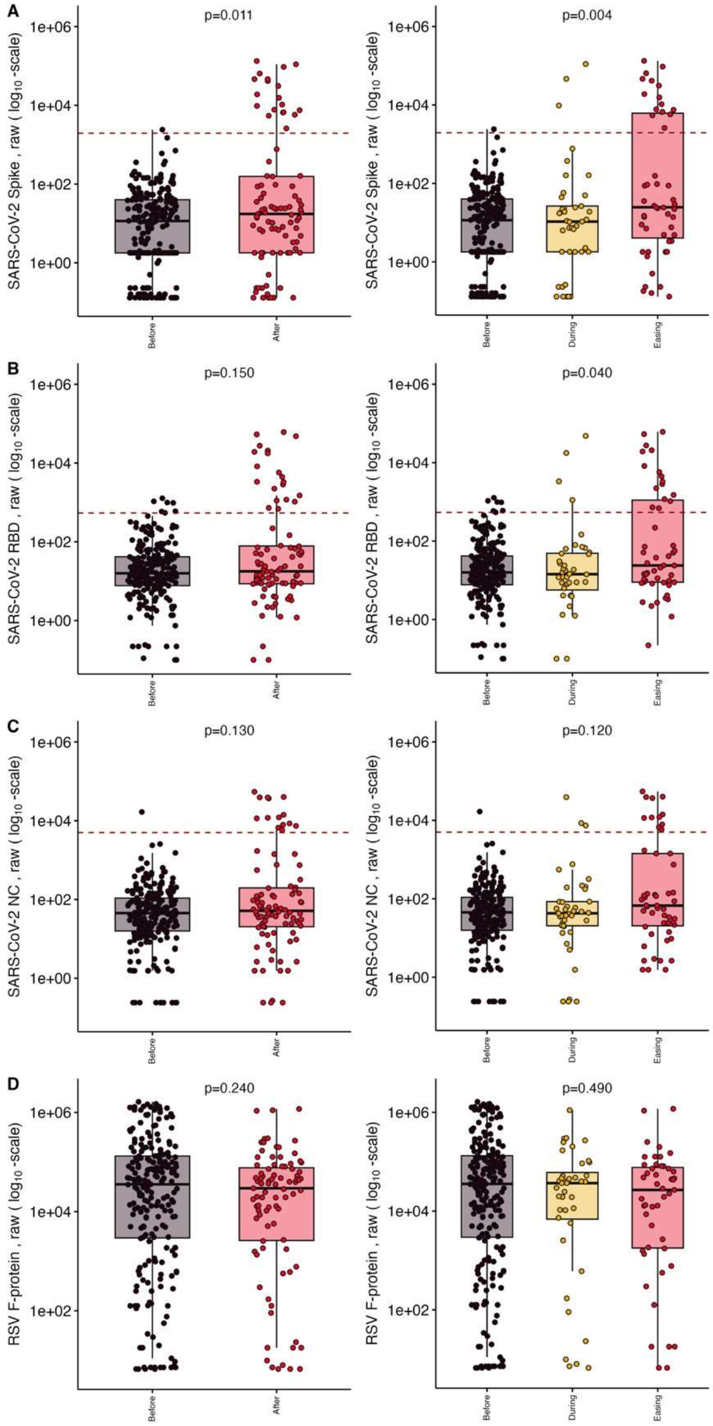

Boxplots show reactivity between before (black) and after (red) March 2020 with differences assessed by a Wilcoxon rank-sum test (left), and before March 2020 (black), during April 2020 to July 2021 (yellow) or after July 2021 (red) with differences assessed using a Kruskal-Wallis test (right) for: **A**. SARS-CoV-2 Spike protein; **B.** SARS-CoV-2 Spike RBD; **C.** SARS-CoV-2 Nucleocapsid protein; and **D**. RSV F-protein, which was representative of

© 2025 Dokal K et al. *JAMA Network Open*.

distributions observed for other viruses. For the SARS-CoV-2 antigens, dashed horizontal lines indicate the manufacturer-recommend threshold for positivity.

© 2025 Dokal K et al. *JAMA Network Open*.

eFigure 9. Reactivity to SARS-CoV-2 Antigens by Age Band

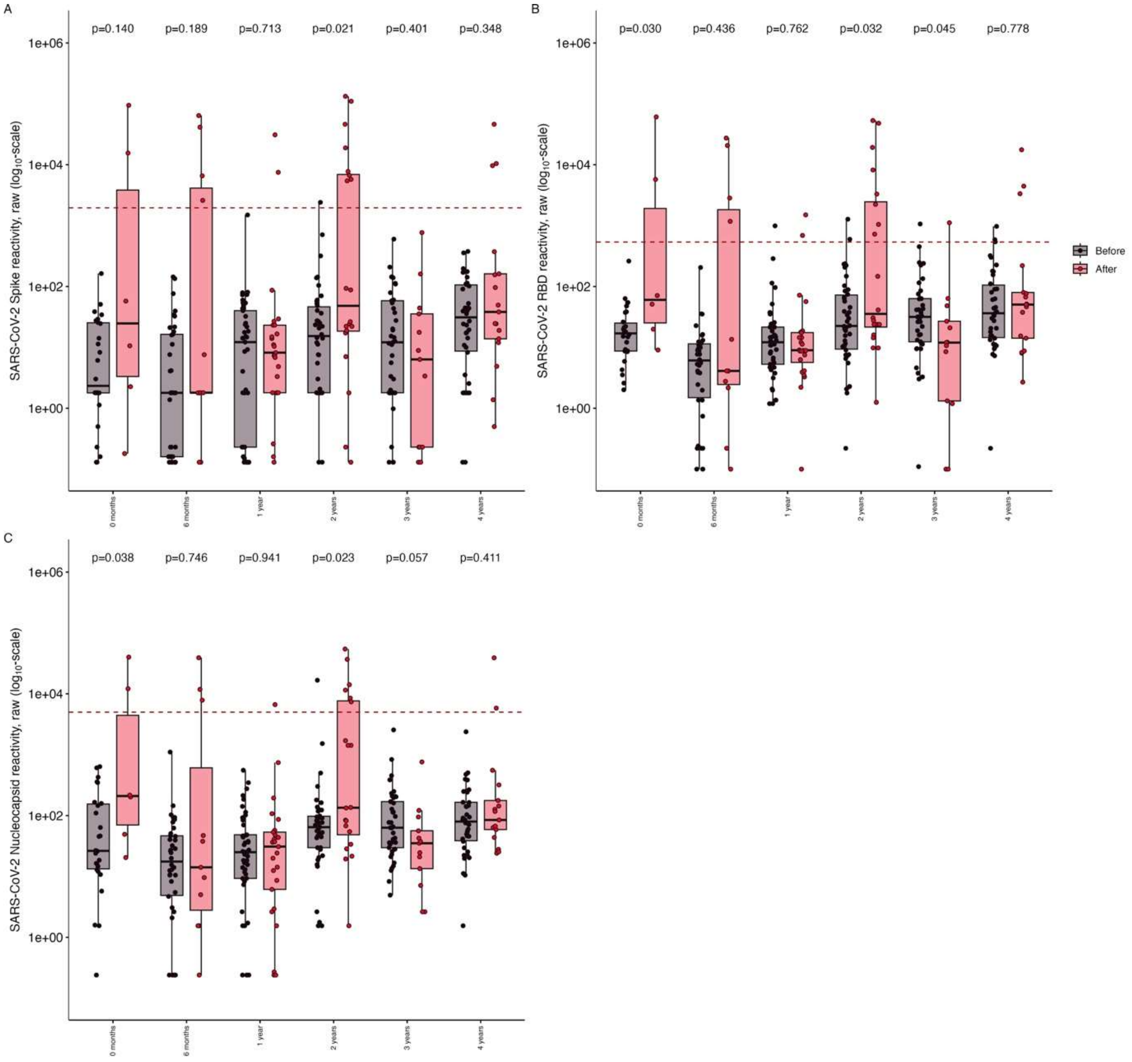

Box plots show reactivity before (black) and after (red) March 2020 with the difference between groups assessed by a Wilcoxon rank-sum test. **A**. SARS-CoV-2 Spike protein; **B.** SARS-CoV-2 Spike RBD; **C.** SARS-CoV-2 Nucleocapsid protein.

© 2025 Dokal K et al. *JAMA Network Open*.

eFigure 10. Changes in *S pyogenes* Disease Incidence by Age

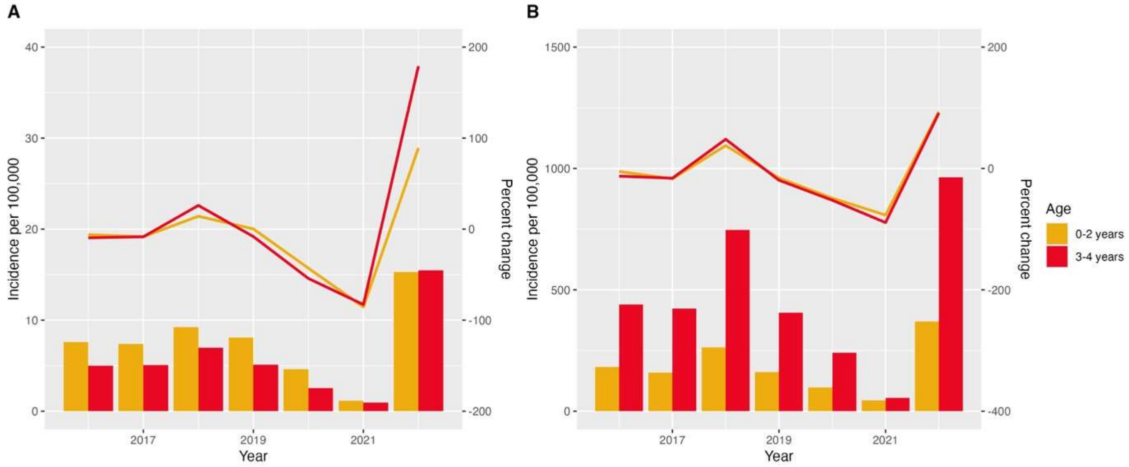

eFigure 10. Changes in *S pyogenes* Disease Incidence by Age Bar charts showing incidence (left axis) per 100,000 person-years with a line indicating the percentage change (right axis) relative to 2016-2019. **A.** iGAS; **B.** Scarlet fever.

© 2025 Dokal K et al. *JAMA Network Open*.

eReferences.

1. Shah P, Voice M, Calvo-Bado L, et al. Relationship between molecular pathogen detection and clinical disease in febrile children across Europe: a multicentre, prospective observational study. *Lancet Reg Health Eur*. 2023;32:100682. doi:10.1016/j.lanepe.2023.100682

2. Calvez R, Taylor A, Calvo-Bado L, Fraser D, Fink CG. Molecular detection of SARS-CoV-2 using a reagent-free approach. *PLoS One*. 2020;15(12):e0243266. doi:10.1371/journal.pone.0243266

3. UK Health Security Agency. Laboratory reporting to UKHSA: A guide for diagnostic laboratories. Published 2023. Available at: https://assets.publishing.service.gov.uk/media/66e2e0ba0d913026165c3d77/UKHSA_Laboratory_reporting_guidelines_May_2023.pdf. Accessed 9th April 2025

4. UK Health Security Agency. Guidelines for the public health management of scarlet fever outbreaks in schools, nurseries and other childcare settings. Published 2023. Available at: https://www.gov.uk/government/publications/scarlet-fever-managing-outbreaks-in-schools-and-nurseries. Accessed 9th April 2025

5. Wang E, Cook D, Hyndman RJ. A New Tidy Data Structure to Support Exploration and Modeling of Temporal Data. *Journal of Computational and Graphical Statistics*. 2020;29(3):466-478. doi:10.1080/10618600.2019.1695624

6. Bauer DF. Constructing Confidence Sets Using Rank Statistics. *Journal of the American Statistical Association*. 1972;67(339):687-690. doi:10.1080/01621459.1972.10481279

7. HM Government, cabinet office. COVID-19 response: summer 2021. Published 2021. Available at: https://www.gov.uk/government/publications/covid-19-response-summer-2021-roadmap. Accessed 9th April 2025.

8. McCaw Z. RNOmni: Rank Normal Transformation Omnibus Test. Published 2017. Available at: https://cran.r-project.org/web//packages/RNOmni/index.html.

© 2025 Dokal K et al. *JAMA Network Open*.

9. Ovadia A, Dalal I. Transient hypogammaglobulinemia of infancy. *LymphoSign Journal*. 2014;01(01):1-9. doi:10.14785/lpsn-2014-0006

10. Wei T, Simko V. R package 'corrplot': Visualization of a Correlation Matrix. Published 2024. Available at: https://cran.r-project.org/web/packages/corrplot/index.html.

11. Wickham H. ggplot2: Elegant Graphics for Data Analysis. Springer-Verlag New York; 2016.

12. Kassambara, A. ggpubr: 'ggplot2' Based Publication Ready Plots. R package version 0.6.0. CRAN; 2023. https://CRAN.R-project.org/package=ggpubr

© 2025 Dokal K et al. *JAMA Network Open*.
