# Supplementary material for: Immunity to Streptococcus pyogenes and Common Respiratory Viruses at Age 0 to 4 Years After COVID-19 Restrictions
Source: JAMA Netw Open. 2025 Oct 15;8(10):e2537808. doi: 10.1001/jamanetworkopen.2025.37808 (PMC12529189; doi:10.1001/jamanetworkopen.2025.37808)
Supplement: Supplement 3. — Nonauthor Collaborators [file jamanetwopen-e2537808-s003.pdf]

Supplemental Online Content: Nonauthor Collaborators
*First name, last name, and suffix (if applicable) are required and will appear in PubMed.

*Group Name(s): PERFORM Consortium, DIAMONDS Consortium							
***First Name and Middle Initial(s)**	***Last Name**	***Suffix (eg, Jr, III)**	Academic Degrees	Institution	Location (city, state/province, country)	Role or Contribution, eg, chair, principal investigator	Group (if more than 1 Group listed in the byline) and/or Subgroup (eg, Steering Committee)
Michael	Levin			Imperial College London			DIAMONDS
Aubrey	Cunnington			Imperial College London			DIAMONDS
Jethro	Herberg			Imperial College London			DIAMONDS
Myrsini	Kaforou			Imperial College London			DIAMONDS
Victoria J.	Wright			Imperial College London			DIAMONDS
Evangelos	Bellos			Imperial College London			DIAMONDS
Claire	Broderick			Imperial College London			DIAMONDS
Samuel	Channon-Wells			Imperial College London			DIAMONDS
Samantha	Cooray			Imperial College London			DIAMONDS
Tisham	De			Imperial College London			DIAMONDS
Giselle	D'Souza			Imperial College London			DIAMONDS
Amedine	Duret			Imperial College London			DIAMONDS
Anikta	Duseja			Imperial College London			DIAMONDS
Leire	Estamiana Ellorrieta			Imperial College London			DIAMONDS
Diego	Estrada-Rivadeneyra			Imperial College London			DIAMONDS
Rachel	Gallassini			Imperial College London			DIAMONDS
Dominic	Habgood-Coote			Imperial College London			DIAMONDS
Shea	Hamilton			Imperial College London			DIAMONDS
Heather	Jackson			Imperial College London			DIAMONDS
James	Kavanagh			Imperial College London			DIAMONDS
Ilana	Keren			Imperial College London			DIAMONDS
Mahdi	Moradi Marjeneh			Imperial College London			DIAMONDS
Stephanie	Menikou			Imperial College London			DIAMONDS
Samuel	Nichols			Imperial College London			DIAMONDS
Ruud	Nijman			Imperial College London			DIAMONDS
Harsita	Patel			Imperial College London			DIAMONDS
Ivana	Pennisi			Imperial College London			DIAMONDS
Oliver	Powell			Imperial College London			DIAMONDS
Ruth	Reid			Imperial College London			DIAMONDS
Priyen	Shah			Imperial College London			DIAMONDS
Ortensia	Vito			Imperial College London			DIAMONDS
Elizabeth	Whittaker			Imperial College London			DIAMONDS
Clare	Wilson			Imperial College London			DIAMONDS
Rebecca	Womersley			Imperial College London			DIAMONDS
Amina	Abdulla			Imperial College London			DIAMONDS
Sarah	Darnell			Imperial College London			DIAMONDS
Sobia	Mustafa			Imperial College London			DIAMONDS
Pantelis	Georgiou			Imperial College London			DIAMONDS
Jesus	Rodriguez-Manzano			Imperial College London			DIAMONDS
Nicolas	Moser			Imperial College London			DIAMONDS
Ivana	Pennisi			Imperial College London			DIAMONDS
Michael	Carter			Evelina London Children's Hospital, Guy's and St Thomas' NHS Foundation Trust; King's College London			DIAMONDS
Paul	Wellman			Evelina London Children's Hospital, Guy's and St Thomas' NHS Foundation Trust; King's College London			DIAMONDS
Shane	Tibby			Evelina London Children's Hospital, Guy's and St Thomas' NHS Foundation Trust; King's College London			DIAMONDS
Jonathan	Cohen			Evelina London Children's Hospital, Guy's and St Thomas' NHS Foundation Trust; King's College London			DIAMONDS

Supplemental Online Content: Nonauthor Collaborators
*First name, last name, and suffix (if applicable) are required and will appear in PubMed.

*First Name and Middle Initial(s)	*Last Name	*Suffix (eg, Jr, III)	Academic Degrees	Institution	Location (city, state/province, country)	Role or Contribution, eg, chair, principal investigator	Group (if more than 1 Group listed in the byline) and/or Subgroup (eg, Steering Committee)
Francesca	Davis			Evelina London Children's Hospital, Guy's and St Thomas' NHS Foundation Trust; King's College London			DIAMONDS
Julia	Kenny			Evelina London Children's Hospital, Guy's and St Thomas' NHS Foundation Trust; King's College London			DIAMONDS
Marie	White			Evelina London Children's Hospital, Guy's and St Thomas' NHS Foundation Trust; King's College London			DIAMONDS
Matthew	Fish			Evelina London Children's Hospital, Guy's and St Thomas' NHS Foundation Trust; King's College London			DIAMONDS
Aislinn	Jennings			Evelina London Children's Hospital, Guy's and St Thomas' NHS Foundation Trust; King's College London			DIAMONDS
Manu	Shankar-Hari			Evelina London Children's Hospital, Guy's and St Thomas' NHS Foundation Trust; King's College London			DIAMONDS
Katy	Fidler			University Hospitals Sussex			DIAMONDS
Dan	Agranoff			University Hospitals Sussex			DIAMONDS
Viven	Richmond			University Hospitals Sussex			DIAMONDS
Matthew	Seal			University Hospitals Sussex			DIAMONDS
Saul	Faust			University Hospital Southampton NHS Foundation trust			DIAMONDS
Dan	Owen			University Hospital Southampton NHS Foundation trust			DIAMONDS
Ruth	Ensom			University Hospital Southampton NHS Foundation trust			DIAMONDS
Sarah	McKay			University Hospital Southampton NHS Foundation trust			DIAMONDS
Diana	Mondo			University Hospital Southampton NHS Foundation trust			DIAMONDS
Mariya	Shaji			University Hospital Southampton NHS Foundation trust			DIAMONDS
Rachel	Schranz			University Hospital Southampton NHS Foundation trust			DIAMONDS
Prita	Rughani			Barts Health NHS Trust			DIAMONDS
Amutha	Anpananthar			Barts Health NHS Trust			DIAMONDS
Susan	Liebeschuetz			Barts Health NHS Trust			DIAMONDS
Anna	Riddell			Barts Health NHS Trust			DIAMONDS
Divya	Divakaran			Barts Health NHS Trust			DIAMONDS
Louise	Han			Barts Health NHS Trust			DIAMONDS
Nosheen	Khalid			Barts Health NHS Trust			DIAMONDS
Ivone	Lancoma-Malcolm			Barts Health NHS Trust			DIAMONDS
Jessica	Schofield			Barts Health NHS Trust			DIAMONDS
Teresa	Simagan			Barts Health NHS Trust			DIAMONDS
Mark	Peters			Great Ormond Street Hospital for Children NHS Foundation Trust			DIAMONDS
Alasdair	Bamford			Great Ormond Street Hospital for Children NHS Foundation Trust			DIAMONDS
Lauran	O'Neill			Great Ormond Street Hospital for Children NHS Foundation Trust			DIAMONDS
Nazima	Pathan			Cambridge Unversity Hospitals NHS Foundation Trust			DIAMONDS
Esther	Daubney			Cambridge Unversity Hospitals NHS Foundation Trust			DIAMONDS
Deborah	White			Cambridge Unversity Hospitals NHS Foundation Trust			DIAMONDS
Melissa	Heightman			University College London Hospitals NHS Foundation Trust			DIAMONDS
Sarah	Eisen			University College London Hospitals NHS Foundation Trust			DIAMONDS
Terry	Segal			University College London Hospitals NHS Foundation Trust			DIAMONDS
Lucy	Wellings			University College London Hospitals NHS Foundation Trust			DIAMONDS
Simon B	Drysdale			St George's University Hospitals NHS Foundation Trust			DIAMONDS
Nicole	Branch			St George's University Hospitals NHS Foundation Trust			DIAMONDS
Lisa	Hamzah			St George's University Hospitals NHS Foundation Trust			DIAMONDS
Heather	Jarman			St George's University Hospitals NHS Foundation Trust			DIAMONDS
Maggie	Nyirenda			Lewisham and Greenwich NHS Trust			DIAMONDS
Lisa	Capozzi			Lewisham and Greenwich NHS Trust			DIAMONDS
Emma	Gardiner			Lewisham and Greenwich NHS Trust			DIAMONDS
Robert	Moots			Liverpool University Hospital NHS Foundation Trust			DIAMONDS
Madga	Nasher			Liverpool University Hospital NHS Foundation Trust			DIAMONDS
Anita	Hanson			Liverpool University Hospital NHS Foundation Trust			DIAMONDS
Michelle	Linforth			Liverpool University Hospital NHS Foundation Trust			DIAMONDS

*First Name and Middle Initial(s)	*Last Name	*Suffix (eg, Jr, III)	Academic Degrees	Institution	Location (city, state/province, country)	Role or Contribution, eg, chair, principal investigator	Group (if more than 1 Group listed in the byline) and/or Subgroup (eg, Steering Committee)
Sean	O'Riordan			Leeds Teaching Hospitals NHS Trust			DIAMONDS
Donna	Ellis			Leeds Teaching Hospitals NHS Trust			DIAMONDS
Akash	Deep			King's College Hospital NHS Foundation Trust			DIAMONDS
Ivan	Caro			King's College Hospital NHS Foundation Trust			DIAMONDS
Fiona	Shackley			Sheffield Children's NHS Foundation Trust			DIAMONDS
Arianna	Bellini			Sheffield Children's NHS Foundation Trust			DIAMONDS
Stuart	Gormley			Sheffield Children's NHS Foundation Trust			DIAMONDS
Samira	Neshat			University Hospitals of Leicester NHS Foundation Trust			DIAMONDS
Barnaby J	Scholefield			Birmingham Women's and Children's Hospital NHS Foundation Trust			DIAMONDS
Ceri	Robbins			Birmingham Women's and Children's Hospital NHS Foundation Trust			DIAMONDS
Helen	Winmill			Birmingham Women's and Children's Hospital NHS Foundation Trust			DIAMONDS
Stéphane C.	Paulus			University of Oxford Partner Children's Hospital, John Radcliffe Hospital, Oxford			DIAMONDS
Andrew J.	Pollard			University of Oxford Partner Children's Hospital, John Radcliffe Hospital, Oxford			DIAMONDS
Mark	Anthony			University of Oxford Partner Children's Hospital, John Radcliffe Hospital, Oxford			DIAMONDS
Sarah	Hopton			University of Oxford Partner Children's Hospital, John Radcliffe Hospital, Oxford			DIAMONDS
Danielle	Miller			University of Oxford Partner Children's Hospital, John Radcliffe Hospital, Oxford			DIAMONDS
Zoe	Oliver			University of Oxford Partner Children's Hospital, John Radcliffe Hospital, Oxford			DIAMONDS
Sally	Beer			University of Oxford Partner Children's Hospital, John Radcliffe Hospital, Oxford			DIAMONDS
Bryony	Ward			University of Oxford Partner Children's Hospital, John Radcliffe Hospital, Oxford			DIAMONDS
Shrijana	Shrestha			University of Oxford, Nepal Site			DIAMONDS
Andrew J.	Pollard			University of Oxford, Nepal Site			DIAMONDS
Meeru	Gurung			University of Oxford, Nepal Site			DIAMONDS
Puja	Amatya			University of Oxford, Nepal Site			DIAMONDS
Bhishma	Pokhrel			University of Oxford, Nepal Site			DIAMONDS
Sanjeev Man	Bijukchhe			University of Oxford, Nepal Site			DIAMONDS
Madhav Chandra	Gautam			University of Oxford, Nepal Site			DIAMONDS
Sarah	Kelly			University of Oxford, Nepal Site			DIAMONDS
Peter	O'Reilly			University of Oxford, Nepal Site			DIAMONDS
Sonu	Shrestha			University of Oxford, Nepal Site			DIAMONDS
Federico	Martinón-Torres			SERGAS Partner			DIAMONDS
Antonio	Salas			SERGAS Partner			DIAMONDS
Fernando Álvez	González			SERGAS Partner			DIAMONDS
Sonia Ares	Gómez			SERGAS Partner			DIAMONDS
Xabier	Bellos			SERGAS Partner			DIAMONDS
Mirian Ben	García			SERGAS Partner			DIAMONDS
Fernando	Viña			SERGAS Partner			DIAMONDS
Sandra	Carnota			SERGAS Partner			DIAMONDS
María José	Curras-Tuala			SERGAS Partner			DIAMONDS
Ana Dacosta	Urbieta			SERGAS Partner			DIAMONDS
Carlos Durán	Suárez			SERGAS Partner			DIAMONDS
Isabel Ferreiros	Vidal			SERGAS Partner			DIAMONDS
Luisa García	Vicente			SERGAS Partner			DIAMONDS
Alberto	Gómez-Carballa			SERGAS Partner			DIAMONDS
Jose	Gómez Rial			SERGAS Partner			DIAMONDS
Pilar	Leboráns Iglesias			SERGAS Partner			DIAMONDS

Supplemental Online Content: Nonauthor Collaborators
*First name, last name, and suffix (if applicable) are required and will appear in PubMed.

***First Name and Middle Initial(s)**	***Last Name**	***Suffix (eg, Jr, III)**	Academic Degrees	Institution	Location (city, state/province, country)	Role or Contribution, eg, chair, principal investigator	Group (if more than 1 Group listed in the byline) and/or Subgroup (eg, Steering Committee)
Narmeen	Mallah			SERGAS Partner			DIAMONDS
Federico	Martinón-Torres			SERGAS Partner			DIAMONDS
Nazareth	Martinón-Torres			SERGAS Partner			DIAMONDS
José María	Sánchez			SERGAS Partner			DIAMONDS
Belén Mosquera	Pérez			SERGAS Partner			DIAMONDS
Jacobo	Pardo-Seco			SERGAS Partner			DIAMONDS
Sara	Pischedda			SERGAS Partner			DIAMONDS
Sara Rey	Vázquez			SERGAS Partner			DIAMONDS
Irene Rivero	Calle			SERGAS Partner			DIAMONDS
Carmen	Rodríguez-Tenreiro			SERGAS Partner			DIAMONDS
Lorenzo	Redondo-Collazo			SERGAS Partner			DIAMONDS
Antonio	Salas			SERGAS Partner			DIAMONDS
Sonia	Serén Fernández			SERGAS Partner			DIAMONDS
Marisol Vilas	Iglesias			SERGAS Partner			DIAMONDS
Enitan D	Carrol			Liverpool Partner			DIAMONDS
Elizabeth	Cocklin			Liverpool Partner			DIAMONDS
Rebecca	Beckley			Liverpool Partner			DIAMONDS
Abbey	Bracken			Liverpool Partner			DIAMONDS
Ceri	Evans			Liverpool Partner			DIAMONDS
Aakash	Khanijau			Liverpool Partner			DIAMONDS
Rebecca	Lenihan			Liverpool Partner			DIAMONDS
Nadia	Lewis-Burke			Liverpool Partner			DIAMONDS
Karen	Newall			Liverpool Partner			DIAMONDS
Sam	Romaine			Liverpool Partner			DIAMONDS
Jennifer	Whitbread			Liverpool Partner			DIAMONDS
Maria	Tsolia			National and Kapodistrian Unversity of Athens			DIAMONDS
Irini	Eleftheriou			National and Kapodistrian Unversity of Athens			DIAMONDS
Nikos	Spyridis			National and Kapodistrian Unversity of Athens			DIAMONDS
Maria	Tambouratzi			National and Kapodistrian Unversity of Athens			DIAMONDS
Despoina	Maritsi			National and Kapodistrian Unversity of Athens			DIAMONDS
Antonios	Marmarinos			National and Kapodistrian Unversity of Athens			DIAMONDS
Marietta	Xagorari			National and Kapodistrian Unversity of Athens			DIAMONDS
Lourida	Panagiota			National and Kapodistrian Unversity of Athens			DIAMONDS
Pefanis	Aggelos			National and Kapodistrian Unversity of Athens			DIAMONDS
Akinosoglou	Karolina			National and Kapodistrian Unversity of Athens			DIAMONDS
Gogos	Charalambos			National and Kapodistrian Unversity of Athens			DIAMONDS
Maragos	Markos			National and Kapodistrian Unversity of Athens			DIAMONDS
Voulgarelis	Michalis			National and Kapodistrian Unversity of Athens			DIAMONDS
Stergiou	Ioanna			National and Kapodistrian Unversity of Athens			DIAMONDS
Marieke	Emonts			Newcastle upon Tyne Hospitals NHS Foundation Trust and Newcastle University			DIAMONDS
Emma	Lim			Newcastle upon Tyne Hospitals NHS Foundation Trust and Newcastle University			DIAMONDS
John	Isaacs			Newcastle upon Tyne Hospitals NHS Foundation Trust and Newcastle University			DIAMONDS
Kathryn	Bell			Newcastle upon Tyne Hospitals NHS Foundation Trust and Newcastle University			DIAMONDS
Stephen	Crulley			Newcastle upon Tyne Hospitals NHS Foundation Trust and Newcastle University			DIAMONDS
Daniel	Fabian			Newcastle upon Tyne Hospitals NHS Foundation Trust and Newcastle University			DIAMONDS

*First Name and Middle Initial(s)	*Last Name	*Suffix (eg, Jr, III)	Academic Degrees	Institution	Location (city, state/province, country)	Role or Contribution, eg, chair, principal investigator	Group (if more than 1 Group listed in the byline) and/or Subgroup (eg, Steering Committee)
Evelyn	Thomson			Newcastle upon Tyne Hospitals NHS Foundation Trust and Newcastle University			DIAMONDS
Diane	Wallia			Newcastle upon Tyne Hospitals NHS Foundation Trust and Newcastle University			DIAMONDS
Caroline	Miller			Newcastle upon Tyne Hospitals NHS Foundation Trust and Newcastle University			DIAMONDS
Ashley	Bell			Newcastle upon Tyne Hospitals NHS Foundation Trust and Newcastle University			DIAMONDS
Fabian J.S	Van der Velden			Newcastle upon Tyne Hospitals NHS Foundation Trust and Newcastle University			DIAMONDS
Geoff	Shenton			Newcastle upon Tyne Hospitals NHS Foundation Trust and Newcastle University			DIAMONDS
Ashley	Price			Newcastle upon Tyne Hospitals NHS Foundation Trust and Newcastle University			DIAMONDS
Owen	Treloar			Newcastle upon Tyne Hospitals NHS Foundation Trust and Newcastle University			DIAMONDS
Daisy	Thomas			Newcastle upon Tyne Hospitals NHS Foundation Trust and Newcastle University			DIAMONDS
Pablo	Rojo			Servicio Madrileño de Salud (SERMAS)			DIAMONDS
Cristina	Epalza			Servicio Madrileño de Salud (SERMAS)			DIAMONDS
Serena	Villaverde			Servicio Madrileño de Salud (SERMAS)			DIAMONDS
Sonia	Márquez			Servicio Madrileño de Salud (SERMAS)			DIAMONDS
Manuel	Gijón			Servicio Madrileño de Salud (SERMAS)			DIAMONDS
Romina	Varchetta			Servicio Madrileño de Salud (SERMAS)			DIAMONDS
Fátima	Machín			Servicio Madrileño de Salud (SERMAS)			DIAMONDS
Laura	Cabello			Servicio Madrileño de Salud (SERMAS)			DIAMONDS
Irene	Hernández			Servicio Madrileño de Salud (SERMAS)			DIAMONDS
Lourdes	Gutiérrez			Servicio Madrileño de Salud (SERMAS)			DIAMONDS
Ángela	Manzanares			Servicio Madrileño de Salud (SERMAS)			DIAMONDS
T.W. (Taco)	Kuijpers			Amsterdam University Medical Center (Amsterdam UMC), University of Amsterdam			DIAMONDS
M. (Martijn)	Van de Kuip			Amsterdam University Medical Center (Amsterdam UMC), University of Amsterdam			DIAMONDS
A.M. (Marceline)	Van Furth			Amsterdam University Medical Center (Amsterdam UMC), University of Amsterdam			DIAMONDS
J.M (Merlijn)	Van den Berg			Amsterdam University Medical Center (Amsterdam UMC), University of Amsterdam			DIAMONDS
Giske	Biesbroek			Amsterdam University Medical Center (Amsterdam UMC), University of Amsterdam			DIAMONDS
Floris	Verkuil			Amsterdam University Medical Center (Amsterdam UMC), University of Amsterdam			DIAMONDS
Carlijn (C.W)	Van der Zee			Amsterdam University Medical Center (Amsterdam UMC), University of Amsterdam			DIAMONDS
Dasja	Pajkrt			Amsterdam University Medical Center (Amsterdam UMC), University of Amsterdam			DIAMONDS
Michael Boele	van Hensbroek			Amsterdam University Medical Center (Amsterdam UMC), University of Amsterdam			DIAMONDS
Dieneke	Schonenberg			Amsterdam University Medical Center (Amsterdam UMC), University of Amsterdam			DIAMONDS
Mariken	Gruppen			Amsterdam University Medical Center (Amsterdam UMC), University of Amsterdam			DIAMONDS
Sietse	Nagelkerke			Amsterdam University Medical Center (Amsterdam UMC), University of Amsterdam			DIAMONDS
Machiel H	Jansen			Amsterdam University Medical Center (Amsterdam UMC), University of Amsterdam			DIAMONDS
Ines	Goetschalckx			Amsterdam University Medical Center (Amsterdam UMC), University of Amsterdam			DIAMONDS
Lorenza	Romani			Bambino Gesù Children's Hospital (Rome-Italy)			DIAMONDS
Maia	De Luca			Bambino Gesù Children's Hospital (Rome-Italy)			DIAMONDS
Sara	Chiurchiù			Bambino Gesù Children's Hospital (Rome-Italy)			DIAMONDS
Costanza	Tripiciano			Bambino Gesù Children's Hospital (Rome-Italy)			DIAMONDS
Stefania	Mercadante			Bambino Gesù Children's Hospital (Rome-Italy)			DIAMONDS
Clementien L	Vermont			ERASMUS MC-Sophia Children's Hospital			DIAMONDS
Henriëtte A.	Moll			ERASMUS MC-Sophia Children's Hospital			DIAMONDS
Dorine M.	Borensztajn			ERASMUS MC-Sophia Children's Hospital			DIAMONDS
Nienke N.	Hagedoorn			ERASMUS MC-Sophia Children's Hospital			DIAMONDS
Chantal	Tan			ERASMUS MC-Sophia Children's Hospital			DIAMONDS
Joany	Zachariasse			ERASMUS MC-Sophia Children's Hospital			DIAMONDS

Supplemental Online Content: Nonauthor Collaborators
*First name, last name, and suffix (if applicable) are required and will appear in PubMed.

*First Name and Middle Initial(s)	*Last Name	*Suffix (eg, Jr, III)	Academic Degrees	Institution	Location (city, state/province, country)	Role or Contribution, eg, chair, principal investigator	Group (if more than 1 Group listed in the byline) and/or Subgroup (eg, Steering Committee)
W.	Dik			ERASMUS MC-Sophia Children’s Hospital			DIAMONDS
Ching-Fen	Shen			Taiwan			DIAMONDS
Dace	Zavadska			Riga Stradins University (Riga, Latvia)			DIAMONDS
Sniedze	Laivacuma			Riga Stradins University (Riga, Latvia)			DIAMONDS
Aleksandra	Rudzate			Riga Stradins University (Riga, Latvia)			DIAMONDS
Diana	Stoldere			Riga Stradins University (Riga, Latvia)			DIAMONDS
Arta	Barzdina			Riga Stradins University (Riga, Latvia)			DIAMONDS
Elza	Barzdina			Riga Stradins University (Riga, Latvia)			DIAMONDS
Sniedze	Laivacuma			Riga Stradins University (Riga, Latvia)			DIAMONDS
Monta	Madelane			Riga Stradins University (Riga, Latvia)			DIAMONDS
Dagne	Gravele			Riga Stradins University (Riga, Latvia)			DIAMONDS
Dace	Svile			Riga Stradins University (Riga, Latvia)			DIAMONDS
Romain	Basmaci			Assistance Publique - Hôpitaux de Paris			DIAMONDS
Noémie	Lachaume			Assistance Publique - Hôpitaux de Paris			DIAMONDS
Pauline	Bories			Assistance Publique - Hôpitaux de Paris			DIAMONDS
Raja Ben	Tkhayat			Assistance Publique - Hôpitaux de Paris			DIAMONDS
Laura	Chériaux			Assistance Publique - Hôpitaux de Paris			DIAMONDS
Juraté	Davoust			Assistance Publique - Hôpitaux de Paris			DIAMONDS
Kim-Thanh	Ong			Assistance Publique - Hôpitaux de Paris			DIAMONDS
Marie	Cotillon			Assistance Publique - Hôpitaux de Paris			DIAMONDS
Thibault	de Groc			Assistance Publique - Hôpitaux de Paris			DIAMONDS
Sébastien	Le			Assistance Publique - Hôpitaux de Paris			DIAMONDS
Nathalie	Vergnault			Assistance Publique - Hôpitaux de Paris			DIAMONDS
Hélène	Sée			Assistance Publique - Hôpitaux de Paris			DIAMONDS
Laure	Cohen			Assistance Publique - Hôpitaux de Paris			DIAMONDS
Alice de	Tugny			Assistance Publique - Hôpitaux de Paris			DIAMONDS
Nevena	Danekova			Assistance Publique - Hôpitaux de Paris			DIAMONDS
Marine	Mommert-Tripon			BioMérieux			DIAMONDS
Karen	Brengel-Pesce			BioMérieux			DIAMONDS
Marko	Pokorn			University Medical Centre Ljubljana, Slovenia			DIAMONDS
Mojca	Kolnik			University Medical Centre Ljubljana, Slovenia			DIAMONDS
Tadej	Avčin			University Medical Centre Ljubljana, Slovenia			DIAMONDS
Tanja	Avramoska			University Medical Centre Ljubljana, Slovenia			DIAMONDS
Natalija	Bahovec			University Medical Centre Ljubljana, Slovenia			DIAMONDS
Petra	Bogovič			University Medical Centre Ljubljana, Slovenia			DIAMONDS
Lidija	Kitanovski			University Medical Centre Ljubljana, Slovenia			DIAMONDS
Mirijam	Nahtigal			University Medical Centre Ljubljana, Slovenia			DIAMONDS
Lea	Papst			University Medical Centre Ljubljana, Slovenia			DIAMONDS
Tina Plankar	Srovin			University Medical Centre Ljubljana, Slovenia			DIAMONDS
Frac	Strle			University Medical Centre Ljubljana, Slovenia			DIAMONDS
Katarina	Vincek			University Medical Centre Ljubljana, Slovenia			DIAMONDS
Michiel	van der Flier			University Medical Center Utrecht, Utrecht, The Netherlands			DIAMONDS
Wim J.E.	Tissing			University Medical Center Utrecht, Utrecht, The Netherlands			DIAMONDS
Rosalie M	Wösten-van			University Medical Center Utrecht, Utrecht, The Netherlands			DIAMONDS
Sebastiaan J	Vastert			University Medical Center Utrecht, Utrecht, The Netherlands			DIAMONDS

Supplemental Online Content: Nonauthor Collaborators
*First name, last name, and suffix (if applicable) are required and will appear in PubMed.

*First Name and Middle Initial(s)	*Last Name	*Suffix (eg, Jr, III)	Academic Degrees	Institution	Location (city, state/province, country)	Role or Contribution, eg, chair, principal investigator	Group (if more than 1 Group listed in the byline) and/or Subgroup (eg, Steering Committee)
Daniel C	Vijlbrief			University Medical Center Utrecht, Utrecht, The Netherlands			DIAMONDS
Louis J.	Bont			University Medical Center Utrecht, Utrecht, The Netherlands			DIAMONDS
Coco R.	Beudeker			University Medical Center Utrecht, Utrecht, The Netherlands			DIAMONDS
Philipp	Agyeman			University of Bern Partner, Inselspital, Bern University Hospital, University of Bern, Switzerland			DIAMONDS
Christoph	Aebi			University of Bern Partner, Inselspital, Bern University Hospital, University of Bern, Switzerland			DIAMONDS
Nina	Schöbi			University of Bern Partner, Inselspital, Bern University Hospital, University of Bern, Switzerland			DIAMONDS
Mariama	Usman			University of Bern Partner, Inselspital, Bern University Hospital, University of Bern, Switzerland			DIAMONDS
Stefanie	Schlüchter			University of Bern Partner, Inselspital, Bern University Hospital, University of Bern, Switzerland			DIAMONDS
Luregn	Schlapbach			University of Zürich Partner, Kinderspital Zürich, University Children's Hospital Zurich			DIAMONDS
Cornelia	Hagmann			University of Zürich Partner, Kinderspital Zürich, University Children's Hospital Zurich			DIAMONDS
Florian	Zapf			University of Zürich Partner, Kinderspital Zürich, University Children's Hospital Zurich			DIAMONDS
Philipp	Baumann			University of Zürich Partner, Kinderspital Zürich, University Children's Hospital Zurich			DIAMONDS
Barbara	Brotschi			University of Zürich Partner, Kinderspital Zürich, University Children's Hospital Zurich			DIAMONDS
Elisa	Zimmermann			University of Zürich Partner, Kinderspital Zürich, University Children's Hospital Zurich			DIAMONDS
Marion	Meier			University of Zürich Partner, Kinderspital Zürich, University Children's Hospital Zurich			DIAMONDS
Kathrin	Weber			University of Zürich Partner, Kinderspital Zürich, University Children's Hospital Zurich			DIAMONDS
Colin	Frink			Micropathology Ltd			DIAMONDS
Marie	Voice			Micropathology Ltd			DIAMONDS
Leo	Calvo-Bado			Micropathology Ltd			DIAMONDS
Michael	Steele			Micropathology Ltd			DIAMONDS
Jennifer	Holden			Micropathology Ltd			DIAMONDS
Andrew	Taylor			Micropathology Ltd			DIAMONDS
Ronan	Calvez			Micropathology Ltd			DIAMONDS
Catherine	Davies			Micropathology Ltd			DIAMONDS
Benjamin	Evans			Micropathology Ltd			DIAMONDS
Jake	Stevens			Micropathology Ltd			DIAMONDS
Peter	Matthews			Micropathology Ltd			DIAMONDS
Kyle	Billing			Micropathology Ltd			DIAMONDS
Werner	Zenz			Medical University of Graz, Austria			DIAMONDS
Alexander	Binder			Medical University of Graz, Austria			DIAMONDS
Benno	Kohlmaier			Medical University of Graz, Austria			DIAMONDS
Daniela S.	Kohlfürst			Medical University of Graz, Austria			DIAMONDS
Nina A.	Schweintzge			Medical University of Graz, Austria			DIAMONDS
Christoph	Zurl			Medical University of Graz, Austria			DIAMONDS
Susanne	Hösele			Medical University of Graz, Austria			DIAMONDS
Piyush G.	Gampawar			Medical University of Graz, Austria			DIAMONDS
Barbara	Kapo			Medical University of Graz, Austria			DIAMONDS
Manuel	Leitner			Medical University of Graz, Austria			DIAMONDS
Lena	Pölz			Medical University of Graz, Austria			DIAMONDS
Alexandra	Rusu			Medical University of Graz, Austria			DIAMONDS
Glorija	Rajic			Medical University of Graz, Austria			DIAMONDS
Bianca	Stoiser			Medical University of Graz, Austria			DIAMONDS
Martina	Strempfl			Medical University of Graz, Austria			DIAMONDS
Manfred G.	Sagmeister			Medical University of Graz, Austria			DIAMONDS
Sebastian	Bauchinger			Medical University of Graz, Austria			DIAMONDS

Supplemental Online Content: Nonauthor Collaborators
*First name, last name, and suffix (if applicable) are required and will appear in PubMed.

*First Name and Middle Initial(s)	*Last Name	*Suffix (eg, Jr, III)	Academic Degrees	Institution	Location (city, state/province, country)	Role or Contribution, eg, chair, principal investigator	Group (if more than 1 Group listed in the byline) and/or Subgroup (eg, Steering Committee)
Martin	Benesch			Medical University of Graz, Austria			DIAMONDS
Astrid	Ceolotto			Medical University of Graz, Austria			DIAMONDS
Ernst	Eber			Medical University of Graz, Austria			DIAMONDS
Siegfried	Gallistl			Medical University of Graz, Austria			DIAMONDS
Harald	Haidi			Medical University of Graz, Austria			DIAMONDS
Almuthe	Hauer			Medical University of Graz, Austria			DIAMONDS
Christa	Hude			Medical University of Graz, Austria			DIAMONDS
Andrea	Kapper			Medical University of Graz, Austria			DIAMONDS
Markus	Keldorfer			Medical University of Graz, Austria			DIAMONDS
Sabine	Löffler			Medical University of Graz, Austria			DIAMONDS
Tobias	Niedrist			Medical University of Graz, Austria			DIAMONDS
Heidemarie	Pilch			Medical University of Graz, Austria			DIAMONDS
Andreas	Pfleger			Medical University of Graz, Austria			DIAMONDS
Klaus	Pfurtscheller			Medical University of Graz, Austria			DIAMONDS
Siegfried	Rödl			Medical University of Graz, Austria			DIAMONDS
Andrea	Skrabi-Baumgartner			Medical University of Graz, Austria			DIAMONDS
Volker	Strenger			Medical University of Graz, Austria			DIAMONDS
Elmar	Wallner			Medical University of Graz, Austria			DIAMONDS
Maike K	Tauchert			Project partner BBMRI-ERIC			DIAMONDS
Ulrich	von Both			LMU Munich Partner (Germany)			DIAMONDS
Laura	Kolberg			LMU Munich Partner (Germany)			DIAMONDS
Patricia	Schmied			LMU Munich Partner (Germany)			DIAMONDS
Ioanna	Mavridi			LMU Munich Partner (Germany)			DIAMONDS
Irene	Alba-Alejandre			LMU Munich Partner (Germany)			DIAMONDS
Katharina	Danhauser			LMU Munich Partner (Germany)			DIAMONDS
Nikolaus	Haas			LMU Munich Partner (Germany)			DIAMONDS
Matthias	Griese			LMU Munich Partner (Germany)			DIAMONDS
Tobias	Feuchtinger			LMU Munich Partner (Germany)			DIAMONDS
Sabrina	Juranek			LMU Munich Partner (Germany)			DIAMONDS
Matthias	Kappler			LMU Munich Partner (Germany)			DIAMONDS
Eberhard	Lurz			LMU Munich Partner (Germany)			DIAMONDS
Esther	Maier			LMU Munich Partner (Germany)			DIAMONDS
Karl	Reiter			LMU Munich Partner (Germany)			DIAMONDS
Carola	Schoen			LMU Munich Partner (Germany)			DIAMONDS
Sebastian	Schroepf			LMU Munich Partner (Germany)			DIAMONDS
Shunmay	Yeung			London School of Hygiene and Tropical Medicine (LSHTM)			DIAMONDS
Manuel	Dewez			London School of Hygiene and Tropical Medicine (LSHTM)			DIAMONDS
David	Bath			London School of Hygiene and Tropical Medicine (LSHTM)			DIAMONDS
Elizabeth	Fitchett			London School of Hygiene and Tropical Medicine (LSHTM)			DIAMONDS
Fiona	Cresswell			London School of Hygiene and Tropical Medicine (LSHTM)			DIAMONDS
Effua	Usuf			Medical Research Council Unit The Gambia at LSHTM			DIAMONDS
Kalifa	Bojang			Medical Research Council Unit The Gambia at LSHTM			DIAMONDS
Anna	Roca			Medical Research Council Unit The Gambia at LSHTM			DIAMONDS
Isatou	Sarr			Medical Research Council Unit The Gambia at LSHTM			DIAMONDS
Momodou	Saidykhan			Medical Research Council Unit The Gambia at LSHTM			DIAMONDS

*First Name and Middle Initial(s)	*Last Name	*Suffix (eg, Jr, III)	Academic Degrees	Institution	Location (city, state/province, country)	Role or Contribution, eg, chair, principal investigator	Group (if more than 1 Group listed in the byline) and/or Subgroup (eg, Steering Committee)
Ebrahim	Ndure			Medical Research Council Unit The Gambia at LSHTM			DIAMONDS
Pedro	Madrigal			European Bioinformatics Institute (EMBL-EBI), United Kingdom			DIAMONDS
Silvie	Fexova			European Bioinformatics Institute (EMBL-EBI), United Kingdom			DIAMONDS
Artur	Sulik			Medical University of Bialystok, Poland			DIAMONDS
Kacper	Toczylowski			Medical University of Bialystok, Poland			DIAMONDS
Dawid	Lewandowski			Medical University of Bialystok, Poland			DIAMONDS
Michael	Levin						PERFORM
Aubrey	Cunnington						PERFORM
Tisham	De						PERFORM
Jethro	Herberg						PERFORM
Myrsini	Kaforou						PERFORM
Victoria	Wright						PERFORM
Lucas	Baumard						PERFORM
Evangelos	Bellos						PERFORM
Giselle	D'Souza						PERFORM
Rachel	Galassini						PERFORM
Dominic	HabgoodCoote						PERFORM
Shea	Hamilton						PERFORM
Clive	Hoggart						PERFORM
Sara	Hourmat						PERFORM
Heather	Jackson						PERFORM
Ian	Maconochie						PERFORM
Stephanie	Menikou						PERFORM
Naomi	Lin						PERFORM
Samuel	Nichols						PERFORM
Ruud	Nijman						PERFORM
Ivonne Pena	Pas						PERFORM
Priyen	Shah						PERFORM
Hannah	Shailes						PERFORM
Ortensia	Vito						PERFORM
Clare	Wilson						PERFORM
Amina	Abdulla						PERFORM
Ladan	Ali						PERFORM
Sarah	Darnell						PERFORM
Rikke	Jorgensen						PERFORM
Sobia	Mustafa						PERFORM
Salina	Persand						PERFORM
Molly	Stevens						PERFORM
Eunjung	Kim						PERFORM
Benjamin	Pierce						PERFORM
Katy	Fidler						PERFORM
Julia	Dudley						PERFORM
Vivien	Richmond						PERFORM
Emma	Tavliavini						PERFORM
Ching-Fen	Shen						PERFORM

Supplemental Online Content: Nonauthor Collaborators
*First name, last name, and suffix (if applicable) are required and will appear in PubMed.

*First Name and Middle Initial(s)	*Last Name	*Suffix (eg, Jr, III)	Academic Degrees	Institution	Location (city, state/province, country)	Role or Contribution, eg, chair, principal investigator	Group (if more than 1 Group listed in the byline) and/or Subgroup (eg, Steering Committee)
Ching-Chuang	Liu						PERFORM
Shih-Min	Wang						PERFORM
Federico	Martinón-Torres						PERFORM
Antonio	Salas						PERFORM
Fernando	Álvez González						PERFORM
Cristina Balo	Farto						PERFORM
Ruth	Barral-Arca						PERFORM
Maria Barreiro	Castro						PERFORM
Xabier	Bello						PERFORM
Mirian Ben	Garcia						PERFORM
Sandra	Carnota						PERFORM
Miriam	Cebey-López						PERFORM
Maria	José Currás-Tuala						PERFORM
Carlos	Durán Suárez						PERFORM
Luisa García	Vicente						PERFORM
Alberto	Gómez-Carballa						PERFORM
Jose Gómez	Rial						PERFORM
Pilar	Leboráns Iglesias						PERFORM
Nazareth	Martinón-Torres						PERFORM
José María	Sánchez						PERFORM
Belén Mosquera	Pérez						PERFORM
Jacobo	Pardo-Seco						PERFORM
Lidia Piñeiro	Rodríguez						PERFORM
Sara	Pischedda						PERFORM
Sara Rey	Vázquez						PERFORM
Irene	Rivero-Calle						PERFORM
Carmen	Rodríguez-Tenreiro						PERFORM
Lorenzo	Redondo-Collazo						PERFORM
Miguel	Sadiki Ora						PERFORM
Antonio	Salas						PERFORM
Sonia	Serén Fernández						PERFORM
Cristina Serén	Trasorras						PERFORM
Marisol Vilas	Iglesias						PERFORM
Dace	Zavadaska						PERFORM
Anda	Balode						PERFORM
Arta	Bārzdiņa						PERFORM
Dārta	Deksne						PERFORM
Dace	Gardovska						PERFORM
Dagne	Grāvele						PERFORM
Ilze	Grope						PERFORM
Anija	Meiere						PERFORM
Ieva	Nokalna						PERFORM
Jana	Pavāre						PERFORM
Zanda	Pučuka						PERFORM
Katrīna	Selecka						PERFORM

Supplemental Online Content: Nonauthor Collaborators
*First name, last name, and suffix (if applicable) are required and will appear in PubMed.

*First Name and Middle Initial(s)	*Last Name	*Suffix (eg, Jr, III)	Academic Degrees	Institution	Location (city, state/province, country)	Role or Contribution, eg, chair, principal investigator	Group (if more than 1 Group listed in the byline) and/or Subgroup (eg, Steering Committee)
Aleksandra	Sidorova						PERFORM
Dace	Svile						PERFORM
Urzula	Nora Urbāne						PERFORM
Effua	Usuf						PERFORM
Kalifa	Bojang						PERFORM
Syed M.A	Zaman						PERFORM
Fatou	Secka						PERFORM
Suzanne	Anderson						PERFORM
Anna	Rocalsatou Sarr						PERFORM
Momodou	Saidykhan						PERFORM
Saffiatou	Darboe						PERFORM
Samba	Ceesay						PERFORM
Umberto	D'alessandro						PERFORM
Henriette A.	Moll						PERFORM
Dorine M.	Borensztajn						PERFORM
Nienke N.	Hagedoorn						PERFORM
Chantal	Tan						PERFORM
Clementien L.	Vermont						PERFORM
Joany	Zachariasse						PERFORM
W.	Dik						PERFORM
Philipp	Agyeman						PERFORM
Luregn J.	Schlapbach						PERFORM
Christoph	Aebi						PERFORM
Verena	Wyss						PERFORM
Mariama	Usman						PERFORM
Eric	Giannoni						PERFORM
Martin	Stocker						PERFORM
Klara M.	Posfay-Barbe						PERFORM
Ulrich	Heininger						PERFORM
Sara Rey	Bernhard-						PERFORM
Anita	Niederer-Loher						PERFORM
Christian	Kahlert						PERFORM
Giancarlo	Natalucci						PERFORM
Christa	Relly						PERFORM
Thomas	Riedel						PERFORM
Christoph	Aebi						PERFORM
Christoph	Berger						PERFORM
Enitan D.	Carrol						PERFORM
Stéphane	Paulus						PERFORM
Elizabeth	Cocklin						PERFORM
Aakash	Khanijau						PERFORM
Rebecca	Jennings						PERFORM
Joanne	Johnston						PERFORM
Simon	Leigh						PERFORM
Karen	Newall						PERFORM

Supplemental Online Content: Nonauthor Collaborators
*First name, last name, and suffix (if applicable) are required and will appear in PubMed.

*First Name and Middle Initial(s)	*Last Name	*Suffix (eg, Jr, III)	Academic Degrees	Institution	Location (city, state/province, country)	Role or Contribution, eg, chair, principal investigator	Group (if more than 1 Group listed in the byline) and/or Subgroup (eg, Steering Committee)
Sam	Romaine						PERFORM
Maria	Tsolia						PERFORM
Irini	Eleftheriou						PERFORM
Maria	Tambouratzi						PERFORM
Antonis	Marmarinos						PERFORM
Marietta	Xagorari						PERFORM
Kelly	Syggelou						PERFORM
Colin	Fink						PERFORM
Marie	Voice						PERFORM
Leo	Calvo-Bado						PERFORM
Werner	Zenz						PERFORM
Benno	Kohlmaier						PERFORM
Nina A.	Schweintzger						PERFORM
Manfred G.	Sagmeister						PERFORM
Daniela S.	Kohlfürst						PERFORM
Christoph	Zurl						PERFORM
Alexander	Binder						PERFORM
Susanne	Hösele						PERFORM
Manuel	Leitner						PERFORM
Lena	Pölz						PERFORM
Glorija	Rajic						PERFORM
Sebastian	Bauchinger						PERFORM
Hinrich	Baumgart						PERFORM
Martin	Benesch						PERFORM
Astrid	Ceolotto						PERFORM
Ernst	Eber						PERFORM
Siegfried	Gallistl						PERFORM
Gunther	Gores						PERFORM
Harald	Haidl						PERFORM
Almuthe	Hauer						PERFORM
Christa	Hude						PERFORM
Markus	Keldorfer						PERFORM
Larissa	Krenn						PERFORM
Heidemarie	Pilch						PERFORM
Andreas	Pfleger						PERFORM
Klaus	Pfurtscheller						PERFORM
Gudrun	Nordberg						PERFORM
Tobias	Niedrist						PERFORM
Siegfried	Rödl						PERFORM
Andrea	Skrabl-Baumgartner						PERFORM
Matthias	Sperl						PERFORM
Laura	Stampfer						PERFORM
Volker	Strenger						PERFORM
Holger	Till						PERFORM
Andreas	Trobisch						PERFORM

Supplemental Online Content: Nonauthor Collaborators
*First name, last name, and suffix (if applicable) are required and will appear in PubMed.

*First Name and Middle Initial(s)	*Last Name	*Suffix (eg, Jr, III)	Academic Degrees	Institution	Location (city, state/province, country)	Role or Contribution, eg, chair, principal investigator	Group (if more than 1 Group listed in the byline) and/or Subgroup (eg, Steering Committee)
Sabine	Löffler						PERFORM
Shunmay	Yeung						PERFORM
Juan	Emmanuel Dewez						PERFORM
Martin	Hibbered						PERFORM
David	Bath						PERFORM
Alec	Miners						PERFORM
Ruud	Nijman						PERFORM
Catherine	Wedderburn						PERFORM
Anne	Meierford						PERFORM
Baptiste	Leurent						PERFORM
Ronald	de Groot						PERFORM
Michiel	Van der Flier						PERFORM
Marien I.	de Jonge						PERFORM
Koen	van Aerde						PERFORM
Wynard	Alkema						PERFORM
Bryan	van den Broek						PERFORM
Jolein	Gloerich						PERFORM
Alain J.	van Gool						PERFORM
Stefanie	Henriet						PERFORM
Martijn	Huijnen						PERFORM
Ria	Philipsen						PERFORM
Esther	Willems						PERFORM
G.P.J.M	Gerrits						PERFORM
M.	van Leur						PERFORM
J.	Heidema						PERFORM
L.	de Haan						PERFORM
C.J.	Miedema						PERFORM
C.	Neeleman						PERFORM
C.C.	Obihara						PERFORM
G.A.	Tramper-Stranders						PERFORM
Andrew J.	Pollard						PERFORM
Rama	Kandasamy						PERFORM
Michael J.	Carter						PERFORM
Daniel	O'Connor						PERFORM
Sagida	Bibi						PERFORM
Dominic F.	Kelly						PERFORM
Meeru	Gurung						PERFORM
Stephen	Thorson						PERFORM
Imran	Ansari						PERFORM
David R.	Murdoch						PERFORM
Shrijana	Shrestha						PERFORM
Zoe	Oliver						PERFORM
Marieke	Emonts						PERFORM
Emma	Lim						PERFORM
Lucille	Valentine						PERFORM

Supplemental Online Content: Nonauthor Collaborators
*First name, last name, and suffix (if applicable) are required and will appear in PubMed.

***First Name and Middle Initial(s)**	***Last Name**	***Suffix (eg, Jr, III)**	Academic Degrees	Institution	Location (city, state/province, country)	Role or Contribution, eg, chair, principal investigator	Group (if more than 1 Group listed in the byline) and/or Subgroup (eg, Steering Committee)
Karen	Allen						PERFORM
Kathryn	Bell						PERFORM
Adora	Chan						PERFORM
Stephen	Crulley						PERFORM
Kirsty	Devine						PERFORM
Daniel	Fabian						PERFORM
Sharon	King						PERFORM
Paul	McAlinden						PERFORM
Sam	McDonald						PERFORM
Anne	McDonnell						PERFORM
Ailsa	Pickering						PERFORM
Evelyn	Thomson						PERFORM
Amanda	Wood						PERFORM
Diane	Wallia						PERFORM
Phil	Woodsford						PERFORM
Frances	Baxter						PERFORM
Ashley	Bell						PERFORM
Matthew	Rhodes						PERFORM
Rachel	Agbeko						PERFORM
Christine	Mackerness						PERFORM
Bryan	Baas						PERFORM
Lieke	Kloosterhuis						PERFORM
Wilma	Oosthoek						PERFORM
Tasnim	Arif						PERFORM
Joshua	Bennet						PERFORM
Kalvin	Collings						PERFORM
Ilona	van der Giessen						PERFORM
Alex	Martin						PERFORM
Aqeela	Rashid						PERFORM
Emily	Rowlands						PERFORM
Gabriella	de Vries						PERFORM
Fabian	van der Velden						PERFORM
Joshua	Soon						PERFORM
Ulrich	von Both						PERFORM
Laura	Kolberg						PERFORM
Manuela	Zwerenz						PERFORM
Judith	Bruschbeck						PERFORM
Christoph	Bidlingmaier						PERFORM
Vera	Binder						PERFORM
Katharina	Danhauser						PERFORM
Nikolaus	Haas						PERFORM
Matthias	Griese						PERFORM
Tobias	Feuchtinger						PERFORM
Julia	Keil						PERFORM
Matthias	Kappler						PERFORM

Supplemental Online Content: Nonauthor Collaborators
*First name, last name, and suffix (if applicable) are required and will appear in PubMed.

***First Name and Middle Initial(s)**	***Last Name**	***Suffix (eg, Jr, III)**	Academic Degrees	Institution	Location (city, state/province, country)	Role or Contribution, eg, chair, principal investigator	Group (if more than 1 Group listed in the byline) and/or Subgroup (eg, Steering Committee)
Eberhard	Lurz						PERFORM
Georg	Muench						PERFORM
Karl	Reiter						PERFORM
Carola	Schoen						PERFORM
François	Mallet						PERFORM
Karen	Brengel-Pesce						PERFORM
Alexandre	Pachot						PERFORM
Marine	Mommert						PERFORM
Marko	Pokorn						PERFORM
Mojca	Kolnik						PERFORM
Katarina	Vincek						PERFORM
Tina Plankar	Srovin						PERFORM
Natalija	Bahovec						PERFORM
Petra	Prunk						PERFORM
Veronika	Osterman						PERFORM
Tanja	Avramoska						PERFORM
Taco	Kuijpers						PERFORM
Ilse	Jongerius						PERFORM
J.M	van den Berg						PERFORM
D.	Schonenberg						PERFORM
A.M.	Barendregt						PERFORM
D.	Pajkrt						PERFORM
M.	van der Kuip						PERFORM
A.M.	van Furth						PERFORM
Evelien	Sprenkeler						PERFORM
Judith	Zandstra						PERFORM
G.	van Mierlo						PERFORM
Judy	Geissler						PERFORM
