# Supplementary material for: Immunity to Streptococcus pyogenes and Common Respiratory Viruses at Age 0 to 4 Years After COVID-19 Restrictions
Source: JAMA Netw Open. 2025 Oct 15;8(10):e2537808. doi: 10.1001/jamanetworkopen.2025.37808 (PMC12529189; doi:10.1001/jamanetworkopen.2025.37808)
Supplement: Supplement 4. — Data Sharing Statement [file jamanetwopen-e2537808-s004.pdf]

Data Sharing Statement

Dokal. Immunity to Streptococcus pyogenes and Common Respiratory Viruses at Age 0 to 4 Years After COVID-19 Restrictions. *JAMA Netw Open*. Published October 15, 2025. doi:10.1001/jamanetworkopen.2025.37808

Data
Data available: Yes
Data types: Data dictionary, Other (please specify)
Additional Information: A subset of the data that support the findings of this study will be made available upon acceptance for publication in a peer-reviewed journal. This will include deidentified reactivity data for M1, M12 and RSV assays as used in our main statistical analysis. Additional deidentified data may be available to bona fide researchers upon reasonable request to the corresponding authors, subject to consideration of ethical restrictions. Complete raw data will not be publicly available due to privacy concerns and ethical restrictions.
How to access data: Email: t.parks@imperial.ac.uk
When available: With publication

Supporting Documents
Document types: None

Additional Information
Who can access the data: A subset of the data that support the findings of this study will be released as an appendix to the main article on acceptance for publication in a peer-reviewed journal. Additional deidentified data may be available to bona fide researchers upon reasonable request.
Types of analyses: Data will be available for the specified purpose of investigating acquisition of immunity to Streptococcus pyogenes and common respiratory viruses.
Mechanisms of data availability: A subset of the data that support the findings of this study will be released as an appendix to the main article on acceptance for publication in a peer-reviewed journal. Additional deidentified data may be available to bona fide researchers on completion of a signed data access agreement.
Any additional restrictions: Complete raw data will not be publicly available due to privacy concerns and ethical restrictions.
